# Clinical and Molecular Correlates of NLRC5 Expression in Patients With Melanoma

**DOI:** 10.3389/fbioe.2021.690186

**Published:** 2021-07-09

**Authors:** Lei Lv, Qinqin Wei, Zhiwen Wang, Yujia Zhao, Ni Chen, Qiyi Yi

**Affiliations:** ^1^Anhui Cancer Hospital, West Branch of the First Affiliated Hospital of USTC, Division of Life Sciences and Medicine, University of Science and Technology of China, Hefei, China; ^2^School of Basic Medical Sciences, Anhui Medical University, Hefei, China

**Keywords:** melanoma, NLRC5, prognosis, immunotherapy, CTLA-4, PD-1

## Abstract

NLRC5 is an important regulator in antigen presentation and inflammation, and its dysregulation promotes tumor progression. In melanoma, the impact of NLRC5 expression on molecular phenotype, clinical characteristics, and tumor features is largely unknown. In the present study, public datasets from the Cancer Cell Line Encyclopedia (CCLE), Gene Expression Omnibus (GEO), The Cancer Genome Atlas (TCGA), and cBioPortal were used to address these issues. We identify that NLRC5 is expressed in both immune cells and melanoma cells in melanoma samples and its expression is regulated by SPI1 and DNA methylation. NLRC5 expression is closely associated with Breslow thickness, Clark level, recurrence, pathologic T stage, and ulceration status in melanoma. Truncating/splice mutations rather than missense mutations in NLRC5 could compromise the expression of downstream genes. Low expression of NLRC5 is associated with poor prognosis, low activity of immune-related signatures, low infiltrating level of immune cells, and low cytotoxic score in melanoma. Additionally, NLRC5 expression correlates with immunotherapy efficacy in melanoma. In summary, these findings suggest that NLRC5 acts as a tumor suppressor in melanoma via modulating the tumor immune microenvironment. Targeting the NLRC5 related pathway might improve efficacy of immunotherapy for melanoma patients.

## Introduction

Melanoma is a lethal form of skin cancer, with more than 320,000 patients diagnosed with melanoma and 57,043 deaths in 2020 globally (Sung et al., 2021). Metastatic melanoma is very aggressive and challenging to treat, although localized melanoma could be treated successfully by surgery (Kudchadkar et al., 2020). Chemotherapy with dacarbazine or temozolomide, the two most commonly used anti-melanoma chemotherapeutic drugs, had a modest benefit to patients. For example, the 5-year survival rate after dacarbazine treatment is only 2–6% (Kim et al., 2010). In recent years, immunotherapy has dramatically improved the efficacy in treating melanoma (Axelrod et al., 2018). For example, CTLA-4 (Cytotoxic T-Lymphocyte Associated Protein 4) and PD-1/PD-L1 (Programed Death 1/Ligand-1)/inhibitors have greatly improved the poor prognosis of metastatic melanoma (Axelrod et al., 2018). Unfortunately, some melanoma patients did not respond to these inhibitors (primary resistance), and a subset of patients might progress after an initial response (acquired resistance) (Gide et al., 2018). Thus, identifying characteristics of the patients for predicting who could benefit from immunotherapy is a key question. It could avoid possible side effects and reduce medical costs for patients who are unsuitable to receive these kinds of treatment. The progress in this aspect is increasing during the last decades. For example, patients with melanoma infiltrated with high tumor-infiltrating CD8^+^ T cells had a better response to anti-PD-1 therapy and a better prognosis (Daud et al., 2016). Dysregulation of MHC class I leads to immunotherapy resistance in melanoma patients (Lee et al., 2020).

MHC molecules, which including class I and II, are the key proteins to present intracellular and exogenous antigens to CD8^+^ cytotoxic T cells. Dysregulation of them allows a cell to evade immune recognition (Dhatchinamoorthy et al., 2021). NLRC5 has been identified to be an important transactivator for MHC class I genes (Yoshihama et al., 2017). It can specifically associate with some DNA-binding proteins to form the enhanceosome that is recruited to the MHC class I promoter and then transactivates the expression of classical MHC class I genes (HLA-A, HLA-B, and HLA-C), non-classical MHC class I gene (HLA-E and HLA-F), and MHC class I related genes (B2M, TAP1, and PSMB9) (Meissner et al., 2010; Neerincx et al., 2012). The expression of NLRC5 correlates positively with the expression of MHC class I genes both in cell lines and tissues (Meissner et al., 2010; Neerincx et al., 2012; Staehli et al., 2012). And NLRC5-deficient mice exhibit reduced expression of MHC class I genes (Biswas et al., 2012; Staehli et al., 2012; Yao et al., 2012). In addition, NLRC5 also contributes to MHC class II gene expression (Neerincx et al., 2014). NLRC5 could effectively regulate the immune response through other ways. For example, NLRC5-deficient cells could not effectively elicit CD8^+^ T-cell activation, along with diminished cytolytic activity to tumor cells (Staehli et al., 2012). NLRC5 could inhibit the NF-κB signaling, thus negatively regulates the inflammatory responses (Cui et al., 2010). Knockdown/knockout of NLRC5 in human/mouse cells inhibited the IL-1β secretion or production (Cui et al., 2010; Yao et al., 2012).

The role of NLRC5 in tumor is controversial and might depend on the specific tumor type. Increased NLRC5 expression has been detected in renal cell carcinoma (Wang et al., 2019), hepatocellular carcinoma (He et al., 2016), gastric cancer (Li et al., 2018), and non-small cell lung cancer (NSCLC) (Li et al., 2015). High NLRC5 expression in NSCLC patients is associated with poor overall survival (Li et al., 2015). However, it has also been reported that NLRC5 expression decreased in several solid tumors, such as melanoma, ovarian cancer, breast cancer, and prostate cancer (Yoshihama et al., 2016; Tang et al., 2020). In particular, the biological function and clinical significance of NLRC5 in melanoma have not been well demonstrated yet.

In this study, by analyzing the expression and the transcriptional regulation of NLRC5 in melanoma from GEO (Gene Expression Omnibus), TCGA (The Cancer Genome Atlas), and cBioPortal database, we investigated the clinical significance of NLRC5 in melanoma. We explored the biological processes (BPs) and HALLMARKs correlating with NLRC5 expression in melanoma through GSEA (Gene set enrichment analysis). In addition, the correlation between NLRC5 expression and infiltrating level of immune cells, especially for CD8^+^ T cells, has been determined. Furthermore, the value of NLRC5 expression in prediction of response to immunotherapy in melanoma was also assessed.

## Materials and Methods

### Gene Expression Analysis in Public Datasets

NLRC5 expression in cell lines was analyzed using the website of CCLE (Cancer Cell Line Encyclopedia)^1^ (Barretina et al., 2012). NLRC5 and PDCD1 (PD-1) expression in single T cells (including CD4^+^ T cells, CD8^+^ T cells, and other kinds of T cells), B cells, NK cells, cancer-associated fibroblasts (CAFs), endothelial cells, macrophages, and malignant melanoma cells were downloaded from GEO (Gene Expression Omnibus dataset) and were analyzed in two melanoma datasets, including GSE72056 and GSE115978 (Tirosh et al., 2016; Jerby-Arnon et al., 2018). The normalized gene expression, GISTIC copy number, DNA methylation, and clinicopathological characteristics of the patients were downloaded from the UCSC Xena website^2^. The normalized mRNA expression data and clinical characteristics of GEO (Gene Expression Omnibus) melanoma datasets, including GSE54467 (Jayawardana et al., 2015), GSE59455 (Budden et al., 2016), GSE22153 (Jonsson et al., 2010), GSE54467 (Jayawardana et al., 2015), GSE53118 (Barter et al., 2014), GSE65904 (Cabrita et al., 2020), GSE33031 (Staber et al., 2013), GSE42440 (Yuki et al., 2013), GSE48344 (Dispirito et al., 2013), and GSE121446 (Chopin et al., 2019) were downloaded from the GEO platform. For the genes with multiple corresponding probes, we took the median expression value of these probes representing gene expression. The gene expression and “Single Cell Derived Immune Cell Signatures” in the “Liu et al., Nat Medicine 2019” dataset were downloaded from the supplementary data of the paper (Liu et al., 2019). The gene expression and clinical characteristics of the “Riaz et al., Cell 2017” dataset were downloaded from GSE91061 and supplementary data of the paper, respectively (Riaz et al., 2017). The gene expression and clinical characteristics of melanoma samples in “Van Allen et al., Science 2015” and “Snyder et al., NEJM 2014” datasets were obtained from cBioPortal (Chan et al., 2015; Van Allen et al., 2015). The gene expression and clinical characteristics of melanoma samples in “Gide et al., Cancer cell 2019” and “Lauss et al., Nat Commun 2017” datasets were obtained from TIDE^3^ (Lauss et al., 2017; Gide et al., 2019; Fu et al., 2020).

### Mutation Profile

The mutation data of NLRC5, expression data of MHC class I genes in TCGA SKCM, and “Liu et al., Nat Medicine 2019” datasets were obtained from cBioPortal. The mutation mapper figures of NLRC5 (Figure 5B and Supplementary Figure 4A) were generated using cBioPortal.

### The Infiltrating Level of Immune Cells

The infiltrating level of immune cells in melanoma samples of TCGA SKCM, GSE54467, GSE59455, and GSE65904 datasets was estimated using the MCP-counter and TIMER algorithm through TIMER2^4^ (Becht et al., 2016; Li et al., 2016; Sturm et al., 2019). The stromal score, immune score, estimate score, and infiltrating level of immune cells in melanoma samples of “Van Allen et al., Science 2015” and “Snyder et al., NEJM 2014” datasets were obtained from cBioPortal.

### Chromatin Immunoprecipitation Sequencing (ChIP-seq) Analysis

Five SPI1 ChIP-seq datasets, including GSM2592808 (Kang et al., 2017), GSM1681426 (Schmidt et al., 2016), GSM1681423 (Schmidt et al., 2016), GSM2359985 (Seuter et al., 2017), and GSM2359987 (Seuter et al., 2017) were used to analyze the binding of SPI1 to NLRC5 promoter. The ChIP-seq peaks were displayed using the Cistrome (Mei et al., 2017).

### Survival Analysis

Correlations between gene expression and patient survival were analyzed by using “survminer” and “survival” packages in R. Auto select optimal cutoff was determined by the R package “survminer” in Figure 2 and Supplementary Figure 3, and the median value was chosen as the cutoff in Figure 10. The cancer samples were split into high and low groups according to the cutoff value. The hazard ratio with 95% confidence intervals and log-rank *p*-value was calculated by R package “survival.”

### Gene Set Enrichment Analysis (GSEA)

Spearman rank correlation coefficients were calculated between the NLRC5 expression and expression of all other genes, which was utilized to create a ranked gene list for GSEA. Then GSEA was performed based on the “biological process” signature (c5.bp.v7.1.symbols.gmt) and HALLMARK signature (h.all.v7.1.symbols.gmt) from MSigDB. Genesets with NOM *p*-val < 0.05, FDR *q*-val < 0.05 and FWER *p*-val < 0.05 were considered significant enrichment terms (Subramanian et al., 2005).

### Protein-Protein Interaction (PPI) Analysis

STRING^5^ was used to retrieve the interacting protein of NLRC5. Then, an enrichment analysis of these proteins for molecular function, biological process, and cellular component was performed using STRING webtool.

### Statistical Analysis

Student’s *t*-test was used to compare the data between two groups. Spearman coefficients were used to correlate gene expression, the infiltrating level of immune cells, and cytotoxic score. Kaplan-Meier analysis using the “survival” and “survminer” package was performed to compare the patients’ survival time between the two groups. Statistical analyses in this study were performed using GraphPad Prism (version 8.3) or R software. *P* values<0.05 were considered statistically significant.

## Results

### The NLRC5 Expression in Melanoma

**FIGURE 1:**
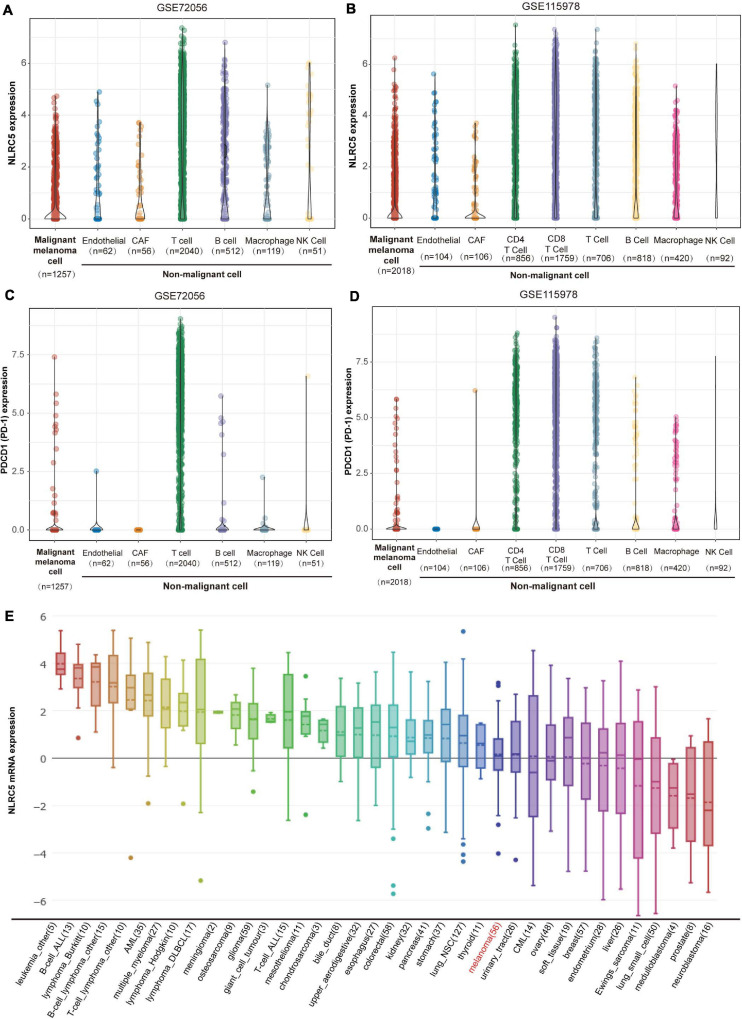
NLRC5 expression in melanoma. **(A)** NLRC5 mRNA expression in single malignant melanoma cells, endothelial cells, CAFs (cancer-associated fibroblasts), T cells, B cells, macrophages, and NK Cells analyzed from melanoma datasets GSE72096. **(B)** NLRC5 mRNA expression in single malignant melanoma cells, endothelial cells, CAFs, CD4^+^ T cells, CD8^+^ T cells, other kinds of T cells, B cells, macrophages, and NK Cells analyzed from melanoma dataset GSE115978. **(C)** PDCD1 (PD-1) mRNA expression in single malignant melanoma cells, endothelial cells, CAFs, T cells, B cells, macrophages, and NK Cells was analyzed from melanoma dataset GSE72056. **(D)** PDCD1 (PD-1) mRNA expression in single malignant melanoma cells, endothelial cells, CAFs, CD4^+^ T cells, CD8^+^ T cells, other kinds of T cells, B cells, macrophages, and NK Cells analyzed from melanoma datasets GSE115978. **(E)** NLRC5 mRNA expression in melanoma cell lines and other kinds of cell lines from the CCLE (Cancer Cell Line Encyclopedia) dataset. The *x*-axis indicates the kind and number of cell lines.

NLRC5 is a key regulator of immune responses (Kobayashi and van den Elsen, 2012). However, whether NLRC5 is expressed only in immune cells or not is unclear in melanoma. Single-cell RNA sequencing analysis from two melanoma datasets, including GSE72056 and GSE115978, showed that NLRC5 is expressed in not only immune cells, including macrophages, NK cells, T cells, and B cells, but also in endothelial cells, CAFs (cancer-associated fibroblasts), and malignant melanoma cells in melanoma samples (Figures 1A,B). In contrast to NLRC5, PD-1, which also regulates immune response, is mainly expressed in T cells rather than in malignant melanoma cells (Figures 1C,D). Additionally, by analyzing the sequencing data from CCLE (Cancer Cell Line Encyclopedia), we showed that NLRC5 is expressed in various cancer cell lines, including melanoma cell lines (Figure 1E). Its expression is highest in immune cells like B-cell ALL (Acute Lymphoblastic Leukemia) cell lines and is lowest in neuroblastoma cell lines (Figure 1E).

Next, we obtained the bulk sequencing data of NLRC5 and clinical datasheet from the TCGA SKCM (Skin Cutaneous Melanoma) dataset and analyzed its clinical significance in melanoma. We classified the enrolled patients into the NLRC5-high group and the NLRC5-low group according to NLRC5 mRNA expression. By evaluating the association between the clinical-pathological characteristics and NLRC5 expression, the clinical-pathological significance of the NLRC5 expression in melanoma was assessed. There was no significant association between NLRC5 expression and sample type, gender, age, pathologic M/N stage, or pathologic stage. However, low NLRC5 expression was associated with thicker Breslow thickness (*p* < 0.0001), higher Clark level (*p* = 0.0003), ulceration (*p* = 0.0003), advanced T stage (*p* = 0.0376) and more new tumor events after initial treatment (*p* = 0.0028) (Table 1).

**TABLE 1:** Characteristics of melanoma patients between NLRC5 low and high groups in TCGA SKCM dataset.

**Characteristic**	**Expression of NLRC5**				***p*–value**
	**Low expression**		**High expression**		
**Age (years)**					0.7064
55	95	41.3%	100	43.3%	
>55	135	58.7%	131	56.7%	
**Sex**					0.7046
Female	88	37.4%	92	39.3%	
Male	147	62.6%	142	60.7%	
**Breslow thickness (mm)**					<0.0001
2.0	51	28.5%	88	49.2%	
>2.0	128	71.5%	91	50.8%	
**Clark level**					0.0003
Level I-III	37	22.3%	64	41.6%	
Level IV-V	129	77.7%	90	58.4%	
**Ulceration**					0.0045
NO	63	38.9%	83	55.3%	
YES	99	61.1%	67	44.7%	
**Pathologic stage**					0.923
Stage 0-II	118	54.9%	120	55.6%	
Stage III-IV	97	45.1%	96	44.4%	
**New tumor event after initial treatment**					0.0028
NO	89	38.7%	123	52.8%	
YES	141	61.3%	110	47.2%	
**Sample type**					0.0953
Primary Tumor	59	25.2%	44	18.7%	
Metastatic	175	74.8%	191	81.3%	
**Pathologic M**					0.0978
M0	204	92.7%	213	96.4%	
M1	16	7.3%	8	3.6%	
**Pathologic N**					0.9208
N0	115	56.7%	120	57.4%	
N1-3	88	43.3%	89	42.6%	
**Pathologic T**					0.0376
T0,Tis	15	7.7%	16	8.1%	
T1-3	93	47.4%	117	59.4%	
T4	88	44.9%	64	32.5%	

**FIGURE 2:**
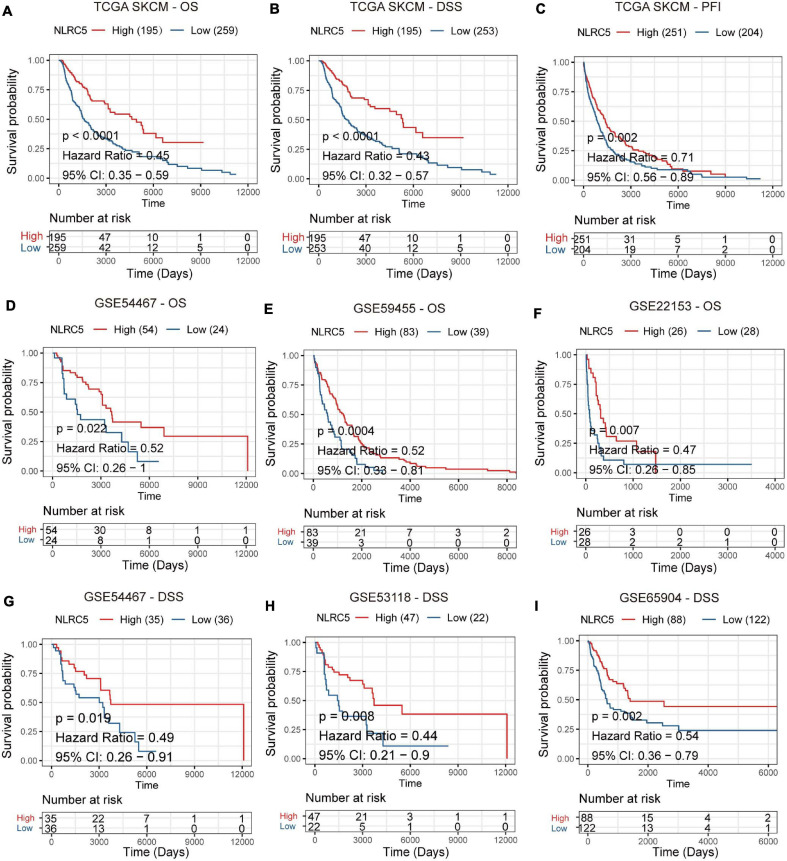
Prognostic value of NLRC5 expression in the melanoma. **(A–C)** Kaplan–Meier analysis of OS (overall survival), DSS (disease-specific survival), and PFI (progression-free interval) of patients with melanoma according to the NLRC5 expression in TCGA SKCM dataset. **(D–F)** Kaplan–Meier analysis of OS (overall survival) of patients with melanoma according to the NLRC5 expression in GSE54467 **(D)**, GSE59455 **(E)**, and GSE22153 **(F)** melanoma datasets. **(G–I)** Kaplan–Meier analysis of DSS (disease-specific survival) of patients with melanoma according to the NLRC5 expression in GSE54467 **(G)**, GSE53118 **(H)**, and GSE65904 **(I)** melanoma datasets, respectively. The patients were stratified into high and low groups using the auto-select best cutoff determined by the R package “survminer.” Red: high NLRC5 expression group, green: low NLRC5 expression group.

Then, we performed Kaplan-Meier analysis of NLRC5 expression in melanoma patients and found that low expression of NLRC5 was significantly associated with poor OS (overall survival) (log-rank *p* < 0.0001, Figure 2A), DSS (disease specific survival) (log-rank *p* < 0.0001, Figure 2B), and PFI (progression-free interval) (log-rank *p* = 0.002, Figure 2C) in TCGA SKCM. The consistent results were further confirmed by Kaplan-Meier Plotter analysis of five GEO datasets. Low expression of NLRC5 was associated with poor OS in GSE54467 (log-rank *p* = 0.022, Figure 2D), GSE59455 (log-rank *p* = 0.0004, Figure 2E), and GSE22153 (log-rank *p* = 0.007, Figure 2F). And it was also associated with DSS in GSE54467 (log-rank *p* = 0.019, Figure 2G), GSE53118 (log-rank *p* = 0.008, Figure 2H), and GSE65904 (log-rank *p* = 0.002, Figure 2I), indicating the close correlation between low expression of NLRC5 and poor outcomes of melanoma patients. Altogether, these results suggest the prognostic value of the reduced expression of NLRC5 in melanoma patients.

### Regulation of NLRC5 Expression

**FIGURE 3:**
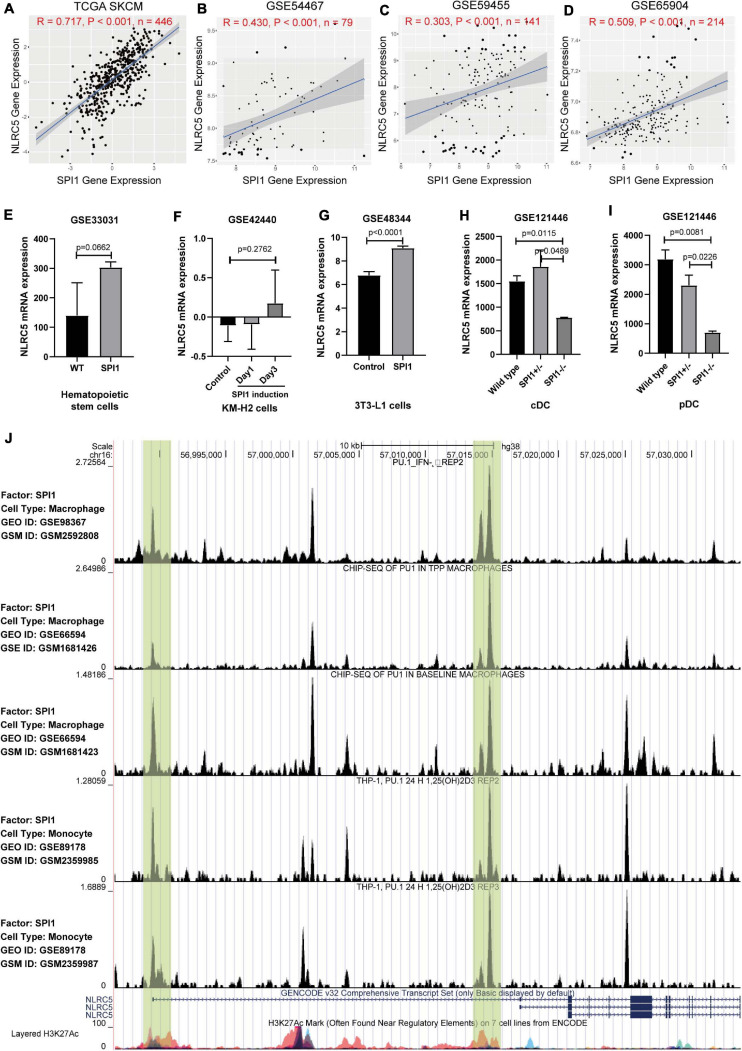
The expression of NLRC5 is regulated by SPI1. **(A–D)** Spearman correlation analysis of NLRC5 expression with SPI1 expression in TCGA SKCM, GSE54467, GSE59455, and GSE65904 melanoma datasets, respectively. Spearman r and *p*-value for each correlation are shown. **(E)** SPI1 knock-in in mouse hematopoietic stem cells (HSCs) promoted NLRC5 expression (reanalysis from GSE33031). **(F)** NLRC5 expression increased after induction of SPI1 in human KM-H2 cells (reanalysis from GSE42440). **(G)** SPI1 overexpression in mouse 3T3-L1 promoted the NLRC5 expression (reanalysis from GSE48344). **(H,I)** Knockout of SPI1 in mouse conventional dendritic cells (cDC) and plasmacytoid dendritic cells (pDC) significantly reduced the NLRC5 expression (reanalysis of GSE121446). **(J)** SPI1 ChIP-seq data in the GEO platform shows that SPI1 binds to the promoter and first intron of NLRC5 gene (Re-analysis of GSE98367, GSE66594, and GSE89178).

Next, we tried to gain insight into the underlying mechanisms of NLRC5 regulation. Correlation analysis between NLRC5 and known transcription factors^6^ expression in TCGA SKCM (Skin Cutaneous Melanoma) showed that SPI1 was one of the transcription factors most related to NLRC5 expression (Supplementary Table 1 and Figure 3A). SPI1 is a central transcription factor in immune response (Carotta et al., 2010; Turkistany and DeKoter, 2011). Single-cell RNA sequencing analysis of GSE72056 and GSE115978 showed that SPI1 is expressed in macrophages, B cells, T cells, and malignant melanoma cells in melanoma samples (Supplementary Figure 1). In addition, we found that NLRC5 expression positively correlated with transcription factor SPI1 expression in 28 kinds of tumor samples from TCGA including TCGA SKCM (Supplementary Table 2 and Figure 3A), which was also consistent with melanoma datasets from the GEO Platform (GSE54467, GSE59455, and GS65904) (Figures 3B–D). Furthermore, correlation analysis in six melanoma immunotherapy datasets showed a highly significant positive correlation between SPI1 and NLRC5 expression (R>0.69, *p* < 0.001, Supplementary Figure 2). Additionally, analysis of GSE33031, GSE42440, GSE48344, and GSE121446 showed that SPI1 overexpression promoted the NLRC5 expression (Figures 3E–G), while analysis from GSE121446 showed that SPI1 knockout resulted in a reduction of NLRC5 expression (Figures 3H,I). Next, we analyzed previously published five SPI1 ChIP-seq datasets (Schmidt et al., 2016; Kang et al., 2017; Seuter et al., 2018) in CistromeDB (Mei et al., 2017; Zheng et al., 2019). In particular, H3K27ac marks the active state of promoters. Analyzation of SPI1 ChIP-seq data revealed that SPI1 binds to the promoter of the NLRC5 gene (Figure 3J). Moreover, Kaplan-Meier analysis of SPI1 expression in melanoma datasets showed that low expression of SPI1 was significantly associated with poor OS (Supplementary Figures 3A,D–F), DSS (Supplementary Figures 3B,G–I), and PFI (Supplementary Figure 3C) in melanoma patients.

**FIGURE 4:**
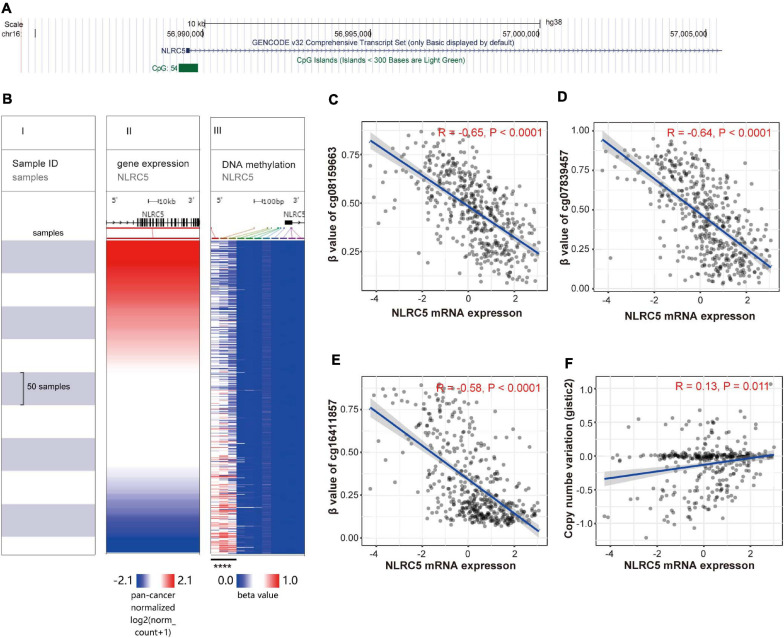
NLRC5 expression correlates with the methylation status of the promoter and copy number variation (CNV). **(A)** A schematic representing the location of NLRC5 in the genome from the UCSC website. **(B)** The heatmap shows the NLRC5 expression and DNA-methylation β value of 11 CpG sites in TCGA SKCM. All samples were ranked from top to bottom according to the NLRC5 expression. Each row is the same sample. (I) samples; (II) NLRC5 expression; (III) β value (methylation level) of each CpG site in the promoter of NLRC5. Blue: low expression/methylation level; Red: high expression/methylation level. The β value of the first three methylation sites negatively correlated with NLRC5 expression (****p < 0.0001). **(C–E)** Spearman correlation analysis of NLRC5 expression with β value of first three methylation sites, including cg08159663, cg07839457, and cg16411857 in TCGA SKCM, respectively. **(F)** Spearman correlation analysis of NLRC5 Copy Number Variation (CNV) with its mRNA expression in TCGA SKCM. Spearman r and *p*-value for each correlation are shown.

DNA methylation and copy number alterations also play important roles in regulating gene expression. We then investigated whether the expression of NLRC5 was regulated by these two factors. Analysis using UCSC Genome Browser showed that there is a CpG island (CGI) located within the promoter of NLRC5 (Figure 4A). Evaluation of the CGI showed that the methylation level of three CpG sites, including cg08159663, cg07839457, and cg16411857, inversely correlated with NLRC5 expression in TCGA SKCM (*R* = −0.65, *p* < 0.0001; *R* = −0.64, *p* < 0.0001; *r* = −0.58, *p* < 0.0001, respectively) (Figures 4B–E). Besides, NLRC5 expression slightly correlated positively with NLRC5 copy number (*R* = 0.13, *p* = 0.011) (Figure 4F).

**FIGURE 5:**
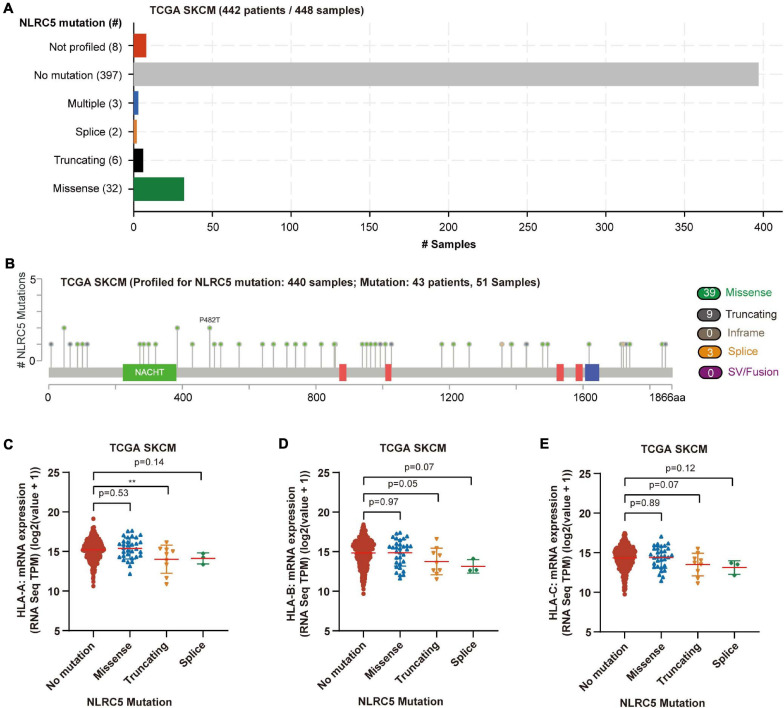
Mutation of NLRC5 in TCGA SKCM. **(A)** Samples with different mutation status of NLRC5 gene. **(B)** Distribution of mutation sites in NLRC5 gene. **(C–E)** Effect of different NLRC5 mutations on HLA-A **(C)**, HLA-B **(D)**, and HLA-C **(E)** expression, respectively. Statistical analysis between every two groups performed by a two-tailed Student’s *t*-test. *P* values were shown as indicated. **p < 0.01.

Next, we analyzed the mutation status of NLRC5 in TCGA SKCM dataset. Among 448 melanoma samples profiled for NLRC5 mutation, missense mutations were found in 32 samples (7.1%), truncating in 6 samples (1.3%), splice in 2 samples (0.4%), and multiple in 3 samples (0.7%) (Figures 5A,B). Among the three samples in multiple group, one sample harbored splice, truncating, and missense mutations of NLRC5, and two samples harbored both truncating and missense mutation. The NLRC5 mutations were randomly distributed across its gene body without obvious hotspots (Figure 5B). To determine whether these mutations affect the protein function, we analyzed its ability to promote the expression of MHC class I genes (HLA-A, HLA-B, and HLA-C), which are downstream genes of NLRC5. The expression levels of all three genes did not show significant differences between the NLRC5 wild type (no mutation) and missense mutation group (Figures 5C–E). However, the expression of these genes in truncating and splice mutation group was lower than that in wild type group, although some of them were not significant because of the small sample number (Figures 5C–E). These results were also confirmed in “Liu et al., Nat Medicine 2019” (Supplementary Figure 4).

Altogether, these findings suggest that NLRC5 expression is modulated positively by the transcriptional regulator SPI1, and possibly negatively regulated by DNA methylation of its promoter. In addition, truncating mutation and splice mutations, but not missense mutations of NLRC5 could compromise the expression of downstream genes in melanoma.

### Low Expression of NLRC5 Correlates With Reduced Immune Infiltration in Melanoma

**FIGURE 6:**
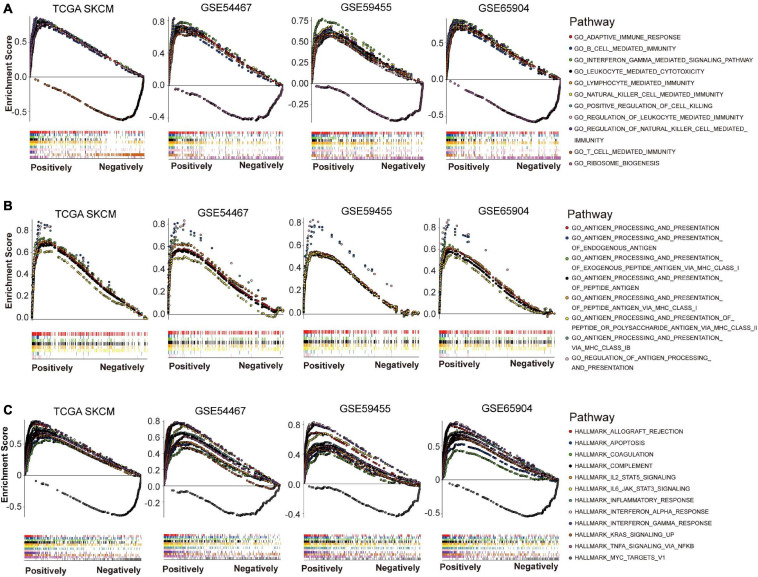
Gene set enrichment analysis (GSEA) of NLRC5 related gene signature in melanoma datasets. **(A)** NLRC5 expression correlated positively with immune-related biological processes (BPs) in TCGA SKCM, GSE54467, GSE59455, and GSE65904 datasets. **(B)** NLRC5 expression correlated positively with biological processes (BPs) implicated in “ANTIGEN PROCESSING AND PRESENTATION” in TCGA SKCM, GSE54467, GSE59455, and GSE65904 datasets. **(C)** The HALLMARK correlating positively/negatively with NLRC5 expression in TCGA SKCM, GSE54467, GSE59455, and GSE65904 datasets.

To explore possible functions that NLRC5 might be involved in melanoma, GSEA was performed using Gene Ontology - biological process (BP) terms in TCGA SKCM and three GEO melanoma datasets (GSE54467, GSE59455, and GSE65904). The common NLRC5 related BPs in all four datasets were used for further analysis. Only one BP, “GO_RIBOSOME_BIOGENESIS,” was significantly negatively associated with NLRC5 expression (Figure 6A and Supplementary Table 3B). Ribosome biogenesis is closely linked to cell division and proliferation (Thomson et al., 2013). This is consistent with the result that the low NLRC5 expression was associated with advanced T stage in melanoma (Table 1). Besides, 235 BPs were significantly positively associated with NLRC5 expression, most of which were immune-associated BPs (Figures 6A,B and Supplementary Table 3A). Particularly, BPs involved in the antigen processing and presentation positively correlated with NLRC5 expression (Figure 6B), consistenting with previous studies that NLRC5 was a pivotal regulator of MHC class I genes, which play key roles in antigen presentation (Cho et al., 2020). Moreover, many other BPs involved in anti-tumor immune response also correlated positively with NLRC5 expression, such as “adaptive immune response,” “B cell mediated immunity,” “leukocyte mediated cytotoxicity,” “lymphocyte mediated immunity,” “T cell mediated immunity,” and “natural killer cell mediated immunity” (Figure 6A and Supplementary Table 3A).

We also used GSEA to explore signaling pathways associated with NLRC5 expression in melanoma by using Gene Ontology HALLMARK terms. The common NLRC5 related HALLMARKs in TCGA SKCM, GSE54467, GSE59455, and GSE65904 were used for further analysis. Only one HALLMARK, that is “MYC TARGETS V1,” was significantly negatively associated with NLRC5 expression (Figure 6C and Supplementary Table 4B). Other 11 HALLMARKs were significantly positively associated with NLRC5 expression (Figure 6C and Supplementary Table 4A), including “INTERFERON GAMMA RESPONSE,” “INTERFERON ALPHA RESPONSE,” “ALLOGRAFT REJECTION,” “IL6 JAK STAT3 SIGNALING,” “INFLAMMATORY RESPONSE,” “COMPLEMENT,” “TNFA SIGNALING VIA NFKB,” “IL2 STAT5 SIGNALING,” “KRAS SIGNALING UP,” “APOPTOSIS,” and “COAGULATION.” Most of these pathways, including “INTERFERON GAMMA RESPONSE,” “INTERFERON ALPHA RESPONSE,” “ALLOGRAFT REJECTION,” “IL6 JAK STAT3 SIGNALING,” “INFLAMMATORY RESPONSE,” “COMPLEMENT,” and “TNFA SIGNALING VIA NFKB,” play important roles in immune regulation. This is consistent with the previous conclusion that NLRC5 expression was positively associated with immune-related BPs. Moreover, we also observed a low activity of apoptosis in low NLRC5 expression melanoma (Figure 6C), suggesting that melanoma with low NLRC5 expression might be chemo/radio-resistant.

**FIGURE 7:**
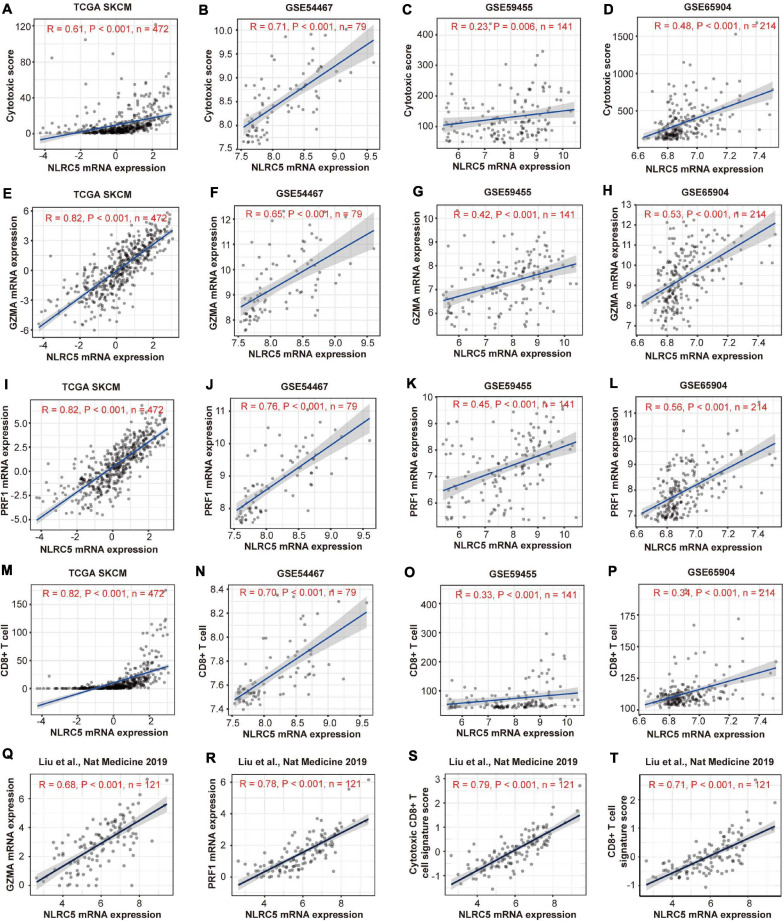
Expressions of NLRC5 correlates positively with cytotoxic signature in melanoma datasets. **(A–D)** Spearman correlation analysis of the NLRC5 expression and cytotoxic score in TCGA SKCM, GSE54467, GSE59455, and GSE65904, respectively. **(E–H)** Spearman correlation analysis of the NLRC5 and GZMA expression in TCGA SKCM, GSE54467, GSE59455, and GSE65904, respectively. **(I–L)** Spearman correlation analysis of the NLRC5 expression and PRF1 expression in TCGA SKCM, GSE54467, GSE59455, and GSE65904, respectively. **(M–P)** Spearman correlation analysis of the NLRC5 expression and infiltrating level of CD8^+^ T cells in TCGA SKCM, GSE54467, GSE59455, and GSE65904, respectively. **(Q–T)** Spearman correlation analysis of the NLRC5 expression and GZMA expression, PRF1 expression, cytotoxic CD8+ T cell signature score, CD8+ T cell signature score in “Liu et al., Nat Medicine 2019,” respectively. Spearman r and *p*-value for each correlation are shown.

Because many genes involved in these immune-related BPs and HALLMARKs are expressed in immune cells, these observations might reflect the infiltrating level of immune cells in melanoma. We then estimated the immune cell infiltration in the above four melanoma datasets using MCP-counter, which provides immune infiltrates’ abundances estimated by immune deconvolution methods (Becht et al., 2016; Li et al., 2020). The analysis revealed that NLRC5 expression was significantly associated with the infiltrating level of T cells, CD8^+^ T cells, NK cells, B cells, monocytes, dendritic cells, and the ratio of macrophage/monocyte, but not neutrophils, endothelial cells, and CAFs (Table 2). TIMER analysis (Li et al., 2017), another immune cell estimation method, also indicated that NLRC5 expression was positively associated with infiltrating level of B cell, CD8^+^ T cell, and Dendritic cell (Supplementary Table 5). We also examined the cytolytic activity (CYT) score of each sample, which is quantified mainly based on the expression of perforin (PRF1) and granzyme A (GZMA) (Rooney et al., 2015). NLRC5 expression was significantly positively associated with both CYT score (Table 2 and Figures 7A–D) and the expression of PRF1 and GZMA in all four melanoma datasets (Figures 7E–L). And the infiltrating level of CD8^+^ T cells, which are predominant cytotoxic T cells expressing GZMA and PRF1 (Farhood et al., 2019), also correlated positively with NLRC5 expression (Figures 7M–P). Furthermore, we analyzed the data of the “Liu et al., Nat Medicine 2019” study, of which both bulk sequencing and single-cell sequencing data are available. Correlation analysis also showed that the expression of NLRC5 positively correlated with the expression of GZMA and PRF1 (Figures 7Q,R), and positively correlated with the abundance of cytotoxic CD8^+^ T cells and CD8^+^ T cells (Figures 7S,T).

**TABLE 2:** Correlation between NLRC5 expression and infiltrating level of immune cell estimated by “MCP-counter” in melanoma datasets.

	**TCGA SKCM**		**GSE54467**		**GSE59455**		**GSE65904**	
	**Spearman r**	***p* value**	**Spearman r**	***p* value**	**Spearman r**	***p* value**	**Spearman r**	***p* value**
T cell	0.5924	<0.0001	0.7312	<0.0001	0.3206	0.0001	0.5066	<0.0001
CD8+ T cell	0.8226	<0.0001	0.7048	<0.0001	0.3313	<0.0001	0.336	<0.0001
NK cell	0.7025	<0.0001	0.6028	<0.0001	0.3372	<0.0001	0.5609	<0.0001
Bcell	0.5371	<0.0001	0.5498	<0.0001	0.2137	0.0109	0.4289	<0.0001
Monocyte	0.6193	<0.0001	0.452	<0.0001	0.3722	<0.0001	0.5017	<0.0001
Macrophage/Monocyte	0.6193	<0.0001	0.452	<0.0001	0.3722	<0.0001	0.5017	<0.0001
Dendritic cell	0.5873	<0.0001	0.4437	<0.0001	0.1965	0.0195	0.3659	<0.0001
Neutrophil	−0.0623	0.1762	−0.0646	0.5712	−0.1031	0.2236	0.0188	0.7843
Endothelial cell	0.0221	0.6322	0.1365	0.2304	0.08427	0.3205	0.2112	0.0019
Cancer associated fibroblast	0.1019	0.0269	0.2043	0.0709	0.2642	0.0015	0.0621	0.3657
Cytotoxicity score	0.6084	<0.0001	0.7084	<0.0001	0.232	0.0056	0.4847	<0.0001

**FIGURE 8:**
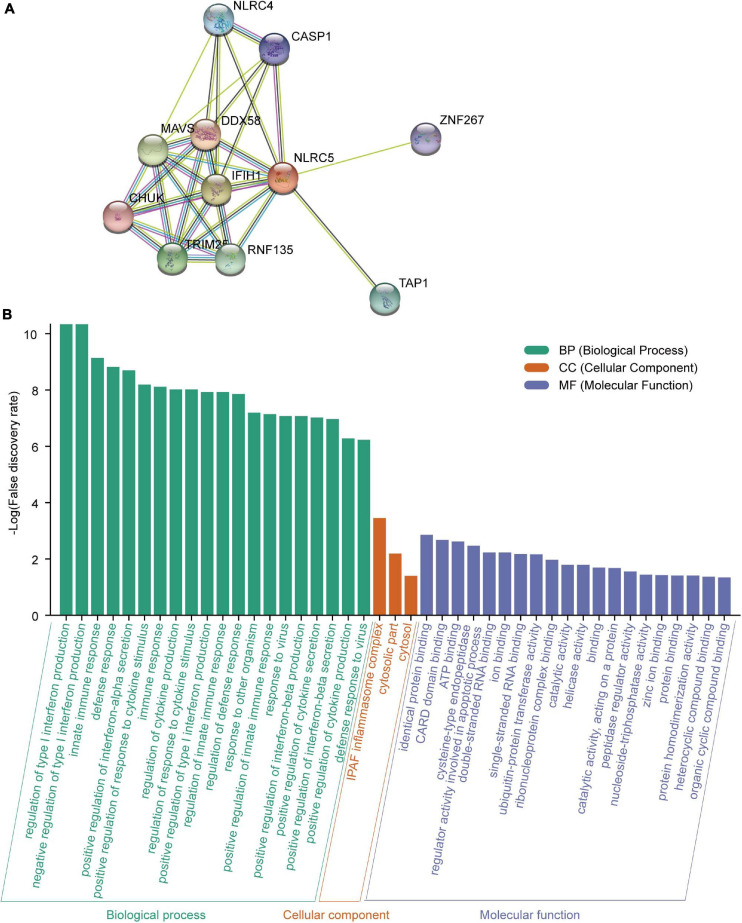
Analysis of the NLRC5 interacting proteins using STRING. **(A)** Protein-protein interaction (PPI) network of NLRC5 visualized by STRING. **(B)** Functional enrichment analysis of NLRC5 interacting proteins in three modules: GO (Biological Process), GO (Molecular Function), and GO (Cellular Component).

Meanwhile, we searched for functional interaction proteins of NLRC5 using the STRING database and found that the top 10 related interaction proteins were IFIH1, ZNF267, TRIM25, RNF135, TAP1, CHUK, DDX58, NLRC4, MAVS, and CASP1 (Figure 8A). GO (Biological Process) analysis showed that these genes were involved in immune-related processes, like “regulation of type I interferon production,” “innate immune response,” “defense response” and “positive regulation of interferon-alpha secretion” (Figure 8B). Among these 11 proteins, seven were involved in “regulation of type I interferon production,” and nine were involved in “innate immune response” (Figure 8B). GO (Molecular Function) analysis showed that these proteins had multiple kinds of binding activity. For example, seven had “identical protein binding” activity (Figure 8B). GO (Cellular Component) analysis showed that two (NLRC4, CASP1) of them interact with NAIP to form the “IPAF inflammasome complex,” which plays a protective role in tumorigenesis (Hu et al., 2010). Moreover, loss of NLRC4 or CASP1 promoted melanoma tumor progression (Hu et al., 2010; Janowski et al., 2016).

Altogether, NLRC5 expression correlates positively with infiltrating level of immune cells in melanoma, especially for CD8^+^ T cells, which are the preferred immune cells for targeting cancer. NRLC5 expression also significantly positively correlates with the expression of cytotoxic genes (PRF1 and GZMA) and cytotoxic score. Moreover, the proteins interacting with NLRC5 are involved in immune-related processes and suppressing tumorigenesis. These results suggest that NLRC5 expression may be a good predictor for tumor immune escape and immunotherapy in melanoma.

### NLRC5 Levels Predict Prognosis and Response to Immunotherapy of Melanoma Patients

As NLRC5 could regulate the expression of MHC genes, and its expression correlated with cytotoxicity and infiltrating level of CD8^+^ T cells in melanoma, we therefore asked whether high NLRC5 expression is associated with good prognosis in patients receiving immunotherapy. We analyzed two datasets, “Van Allen et al., Science 2015” and “Snyder et al., NEJM 2014,” derived from two clinical trials of anti-CTLA4 therapy for metastatic melanoma patients (Snyder et al., 2014; Van Allen et al., 2015).

**FIGURE 9:**
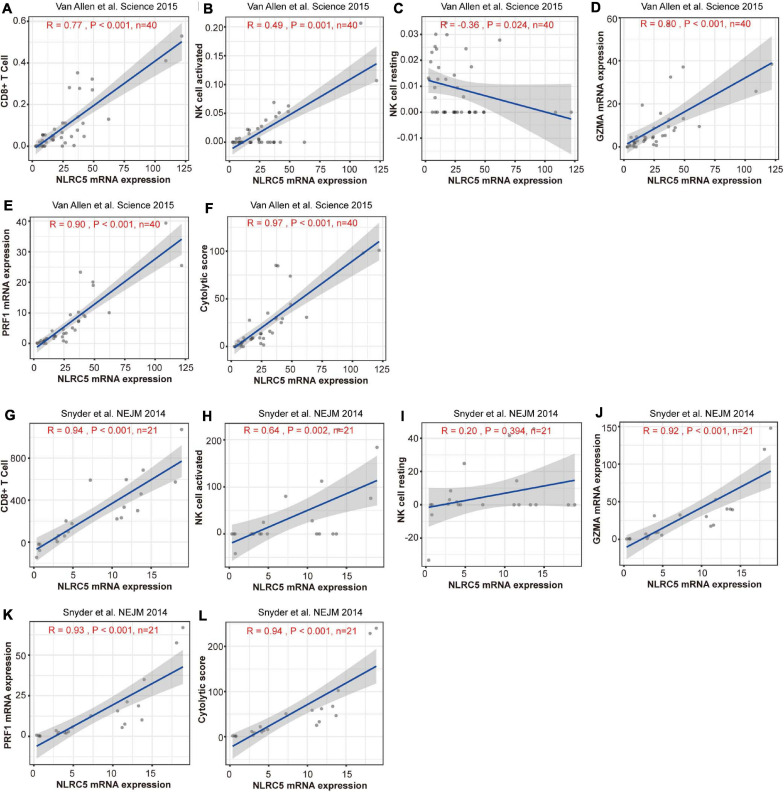
Expression of NLRC5 and cytotoxic signature are positively correlated in melanoma patients receiving immunotherapy. **(A,G)** Spearman correlation analysis of the NLRC5 expression and infiltrating level of CD8^+^ T cells in “Snyder et al. NEJM 2014” and “Snyder et al., NEJM 2014” datasets, respectively. **(B,H)** Spearman correlation analysis of NLRC5 expression and infiltrating level of activated NK cells in “Snyder et al. NEJM 2014” and “Snyder et al., NEJM 2014” datasets, respectively. **(C,I)** Spearman correlation analysis of NLRC5 expression and infiltrating level of resting NK cells in “Snyder et al. NEJM 2014” and “Snyder et al., NEJM 2014” datasets, respectively. **(D,J)** Spearman correlation analysis of NLRC5 and GZMA expression in “Snyder et al. NEJM 2014” and “Snyder et al., NEJM 2014” datasets, respectively. **(E,K)** Spearman correlation analysis of NLRC5 and PRF1 expression in “Snyder et al. NEJM 2014” and “Snyder et al., NEJM 2014” datasets, respectively. **(F,L)** Spearman correlation analysis of the NLRC5 expression and cytotoxic score in “Snyder et al. NEJM 2014” and “Snyder et al., NEJM 2014” datasets, respectively.

Stromal score, immune score, and estimate score, which indicate infiltrating level of stromal cells, immune cells, and tumor purity, respectively, correlated positively with NLRC5 expression in both datasets (Supplementary Figure 5). Further analysis showed that both the infiltrating level of CD8^+^ T cells and “activated NK cells” correlated positively with NLRC5 expression, but not for “resting NK cells” (Figures 9A–C,G–I, Table 3). We also found that NLRC5 expression correlated positively with cytotoxic activity (Figures 9F,L) and expression of cytotoxic genes (GZMA and PRF1) (Figures 9D,E,J,K), which is associated with antitumor immune responses and improved prognosis (Rooney et al., 2015).

**TABLE 3:** Correlation between NLRC5 expression and infiltrating level of immune cell in melanoma receiving immunotherapy.

	**Van Allen et al.,**		**Snyder et al.,**	
	**Science 2015**		**NEJM 2014**	
	**Spearman r**	***p* value**	**Spearman r**	***p* value**
B cell naive	0.4493	0.0036	0.6262	0.0024
B cell memory	−0.02094	0.8979	0.4549	0.0383
B cell plasma	0.6508	<0.0001	0.7932	<0.0001
Tcell CD8+	0.7714	<0.0001	0.9442	<0.0001
Tcell CD4+ naive	0.1076	0.5086		
T cell CD4+ memory resting	−0.1354	0.405	0.4498	0.0408
T cell CD4+ memory activated	0.4736	0.002	0.9129	<0.0001
Tcell follicular helper	0.6615	<0.0001	0.8416	<0.0001
Tcell regulatory (Tregs)	0.2575	0.1087	0.557	0.0087
T cell gamma delta	0.2216	0.1694	0.8054	<0.0001
NK cell resting	−0.3554	0.0244	0.1964	0.3935
NK cell activated	0.4905	0.0013	0.6361	0.0019
Monocyte	0.5722	0.0001	0.6893	0.0005
Macrophage M0	0.2885	0.071	0.8078	<0.0001
Macrophage M1	0.7354	<0.0001	0.8455	<0.0001
Macrophage M2	0.6256	<0.0001	0.8364	<0.0001
Myeloid dendritic cell resting	0.03045	0.852	0.5484	0.0101
Myeloid dendritic cell activated	0.1397	0.3898	0.05096	0.8263
Mast cell activated	−0.05286	0.746	0.5375	0.012
Mast cell resting	0.2335	0.1471	0.3686	0.1001
Eosinophil	−0.3898	0.0129	0.1216	0.5996
Neutrophil	0.2566	0.1099	0.09442	0.6839

**FIGURE 10:**
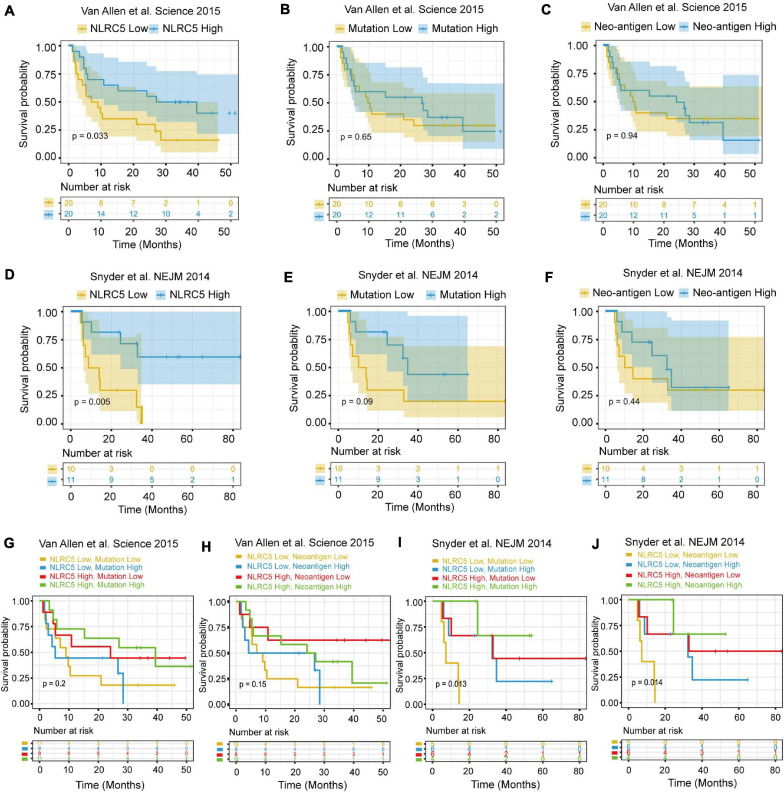
Survival analysis of melanoma patients receiving immunotherapy. **(A–C)** Kaplan–Meier analysis of OS (overall survival) of patients with melanoma according to the NLRC5 expression, mutation load, and neoantigen load in the “Van Allen et al. Science 2015” dataset, respectively. **(D–F)** Kaplan–Meier analysis of OS (overall survival) of patients with melanoma according to the NLRC5 expression, mutation load, and neoantigen load in the “Snyder et al. NEJM 2014” dataset, respectively. **(G)** Kaplan–Meier analysis of OS of patients with melanoma according to both the NLRC5 expression and mutation load in the “Van Allen et al. Science 2015” dataset. The patients were stratified into four groups using the median NLRC5 expression and mutation load as a cutoff. **(H)** Kaplan–Meier analysis of OS of patients with melanoma according to both the NLRC5 expression and neoantigen load in the “Van Allen et al. Science 2015” dataset. The patients were stratified into four groups using the median NLRC5 expression and neoantigen load as a cutoff. **(I,J)** The survival analyses presented were generated as in **(G)** and **(H)** with the “Snyder et al. NEJM 2014” dataset.

We then examined the survival of melanoma patients receiving immunotherapy after dividing the patients into two equal groups (high and low NLRC5 expression). Meanwhile, two previously reported predictors of survival after immunotherapy, mutation-load (the number of point mutations) and neo-antigen load (Chan et al., 2015; Van Allen et al., 2015), were also been included for comparison. We found that patients with low NLRC5 expression had a significantly poorer prognosis than those with high NLRC5 expression in the “Van Allen et al., Science 2015” dataset (*p* = 0.033; Figure 10A). No prognostic significance was found for mutation–load (*p* = 0.65) or neo-antigen (*p* = 0.94) in the “Van Allen et al., Science 2015” dataset (Figures 10B,C). A similar result was found in the “Snyder et al., NEJM 2014” dataset (Figures 10D–F). In addition, no significant correlation between the NLRC5 expression and the mutation/neo-antigen was found in these two melanoma datasets (Supplementary Figure 6). Then, we tested the predictive capacity of the combination of NLRC5 expression and mutation/neo-antigen load in immunotherapy. In the “Snyder et al., NEJM 2014” dataset, patients with low NLRC5 expression and low mutation load/neo-antigen load had the worst prognosis, while patients with high expression of NLRC5 and high mutation load/neo-antigen load had the best prognosis (Figures 10G,H). Similar results were also found from analysis of “Van Allen et al., Science 2015” dataset (Figures 10I,J).

**FIGURE 11:**
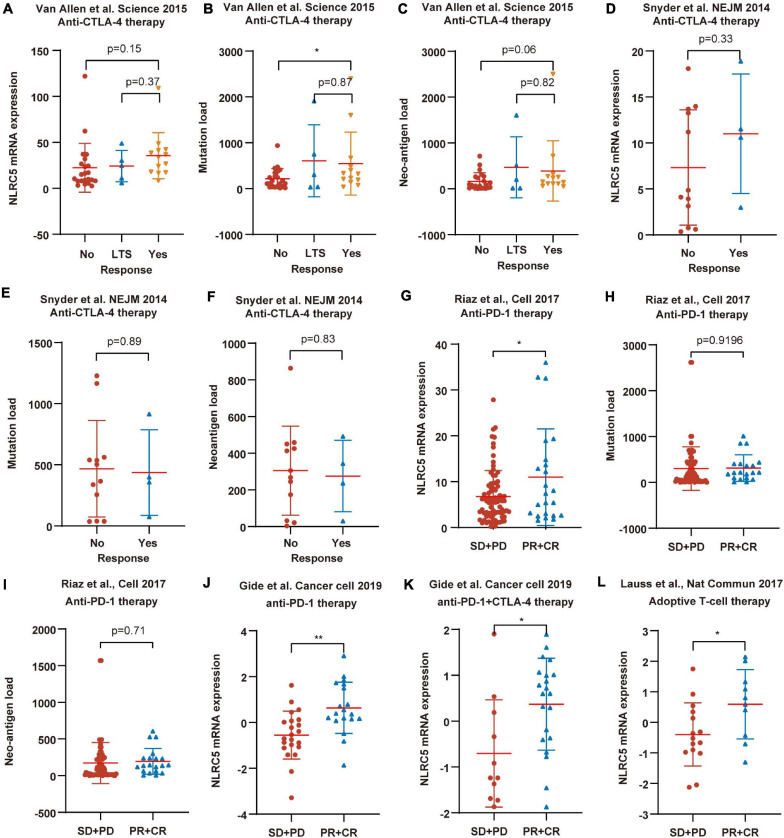
NLRC5 expression is associated with immunotherapy response. **(A)** Low NLRC5 expression is associated with the decreased immunotherapy response rate in “Van Allen et al., Science 2015.” **(B)** Low mutation load is associated with the decreased immunotherapy response rate in “Van Allen et al., Science 2015.” **(C)** Low neo-antigen load is associated with the decreased immunotherapy response rate in “Van Allen et al., Science 2015.” **(D)** Low NLRC5 expression is associated with the decreased immunotherapy response rate in “Snyder et al., NEJM 2014.” **(E,F)** Comparison of mutation load/neo-antigen between response and non-response groups in “Snyder et al., NEJM 2014” dataset. **(G)** NLRC5 expression is lower in the “SD+PD” group than in the “PR+CR” group in “Riaz et al., Cell 2017”. **(H,I)** Comparison of mutation load/neo-antigen between “SD+PD” and “PR+CR” group in “Riaz et al., Cell 2017.” **(J–L)** NLRC5 expression is lower in the “SD+PD” group than in the “PR+CR” group in “Gide et al. Cancer cell 2019” and “Lauss et al., Nat Commun 2017.” The type of immunotherapy received by melanoma patients in each dataset is shown. LTS, Long-term survival with no clinical benefit. RECIST categories: CR, complete response; PD, progressive disease; PR, partial response; SD, stable disease. Statistical analysis between every two groups performed by a two-tailed Student’s *t*-test. *P*-values were shown as indicated. *p < 0.05, **p < 0.01.

To further test the correlation of NLRC5 expression with immunotherapy response, we analyzed more datasets in which patients received different types of immunotherapy. Low NLRC5 expression was associated with decreased response rate to anti-CTLA-4 therapy in both “Van Allen et al., Science 2015” and “Snyder et al., NEJM 2014” (Figures 11A,D). Although the mutation load and neo-antigen load in the non-response group was lower than in the response group of “Van Allen et al., Science 2015” (Figures 11B,C), it was not the case for “Snyder et al., NEJM 2014”(Figures 11E,F). Analysis from “Riaz et al., Cell 2017” showed that NLRC5 expression was lower in the “PD+SD” group than in the “PR+CR” group, indicating the association of low NLRC5 expression with a worse outcome to anti-PD-1 therapy (Figure 11G). However, there were no significant different mutation load/neo-antigen load between the “PD+SD” and “PR+CR” group in “Riaz et al., Cell 2017” (Figures 11H,I). Furthermore, NLRC5 expression was also lower in the “SD+PD” group than in the “PR+CR” group in “Gide et al. Cancer cell 2019” (Figures 11J,K) and “Lauss et al., Nat Commun 2017” (Figure 11L), in which patients receiving anti-PD-1/anti-PD-1+CTLA-4 therapy and adoptive T-cell therapy, respectively.

Overall, these results suggest that, other than mutation load and neo-antigen load, these results suggest that NLRC5 expression may be a more promising predictive biomarker for response to immunotherapy in melanoma.

## Discussion

In this study, we revealed that NLRC5 is expressed in both immune cells and melanoma cells in melanoma samples and that its expression was associated with clinical characteristics such as ulceration status, T stages, recurrence, and prognosis. Its expression was regulated by transcription factor SPI1, and probably negatively regulated by DNA methylation of its promoter. Truncating/splice mutations but not missense mutation of NLRC5 compromise the expression of MHC class I genes. Furthermore, NLRC5 expression correlated positively with the infiltrating level of CD8^+^ T cells and CYT score. At last, melanoma patients with low NLRC5 expression have substantially worse response rates and outcomes to immunotherapy.

NLR (NOD-like receptors) family proteins are cytoplasmic pattern-recognition receptors, playing an essential role in immunity (Dolasia et al., 2018). NLRC5 is one of the largest members of the NLRs family. It promotes the expression of MHC gene expression and thus could be a target for cancer immune evasion (Yoshihama et al., 2016). As MHC class I genes are important to present tumor neo-antigen to CD8^+^ T cells, it implies a tumor-suppressing function of NLRC5. NLRC5 expression in multiple tumors, including prostate, lung, uterine, melanoma, and thyroid cancer, was lower than corresponding normal tissues (Yoshihama et al., 2016). However, NLRC5 promoted tumor malignancy in hepatocellular carcinoma and clear cell renal cell carcinoma (He et al., 2016; Peng et al., 2016; Wang et al., 2019). In this study, we have shown that low NLRC5 expression was associated with multiple worse clinical characteristics and prognosis in melanoma. This is consistent with its function of inducing the expression of MHC-I genes, whose loss often leads to tumor immune escape (Angell et al., 2014; Aptsiauri et al., 2018). We hypothesize that the decreased expression of NLRC5 led to reduced expression of MHC genes in melanoma, which would impair the ability of antigen processing and presentation of tumor cells, leading to tumor immune evasion and thus promoting tumorigenesis in melanoma.

Consistent with a previous study (Yoshihama et al., 2016), the expression of NLRC5 was regulated by its copy number and DNA methylation status of its promoter. Additionally, we also found that the expression of NLRC5 was regulated by SPI1 (PU.1), a master transcription factor in immune cells. The alteration of these factors would influence the expression and function of NLRC5, leading to abnormality of downstream genes, such as HLA-A/B/C. Furthermore, mutation of NLRC5 was also observed in ∼10% of melanoma patients. There was no hotspot for NLRC5 mutation in melanoma. Truncating/splice mutations, but not missense mutations could reduce the expression of HLA-A, HLA-B, and HLA-C. NLRC5 protein consists of three domains, including the N-terminal CARD region, C-terminal receptor domain, and the central NACHT domain (Meissner et al., 2010). It has been reported that the N-Terminal of NLRC5 is responsible for transcriptionally regulating the expression of MHC Class I genes. However, we found missense mutations at the N-terminal of NLRC5 in only 2/440 samples. And both NLRC5 and its downstream MHC Class I genes expression in these two patients were not lower than the mean expression of other patients (data not shown). This indicated that missense mutations of NLRC5 do not seem to impair its ability to mediate the expression of downstream genes in melanoma.

CD8^+^ T-cells express TCRs (T-cell receptors) and recognize the MHC molecules on tumor cells. Then the T cells can kill these tumor cells. As the regulator of MHC class I genes, NLRC5 could enable tumor cells to activate CD8^+^ T cells (Rodriguez et al., 2016; Lupfer et al., 2017), and its deficiency would impair tumor cell killing by CD8^+^ T cells, then promote tumor growth and metastasis (Staehli et al., 2012; Rodriguez et al., 2016). We found that NLRC5 expression correlated positively with infiltrating level of CD8^+^ T cells, suggesting that NLRC5 not only function in activating CD8^+^ T cells but also may function on expanding or recruiting CD8^+^ T cells. Yao et al. found that the CD8^+^ T cells in spleen and liver profoundly decreased in NLRC5^–/–^ mice infected by Listeria monocytogenes (LM), and NLRC5 knockout impaired the CD8^+^ T cell activation in mice infected by LM (Yao et al., 2012). This suggested that NLRC5 played an important role in CD8^+^ T cell expansion during intracellular pathogen infection. Moreover, NLRC5 knockdown significantly reduced the IL-1β secretion of human myeloid cells and primary monocytes during infection (Davis et al., 2011). And NLRC5 knockout in mice also reduced IL-1β production of BMDMs (Bone-marrow-derived macrophages) (Yao et al., 2012). IL-1 has a key role in tissue localization, expansion, effector function, and memory response of CD8^+^ T cells (Davis et al., 2011). This may explain why NLRC5 can activate, expand and recruit CD8^+^ T cells. However, it needs to be validated experimentally.

Immunotherapy through Immune checkpoint blockade (ICB) has significantly improved the outcomes for patients with melanoma and some other advanced cancers (Luke et al., 2017; Queirolo et al., 2019). However, ICB treatment is only effective in a subset of patients with melanoma, so clinical methods have been developed to identify which patients can benefit from these treatments. As mutation of cell surface protein will lead to neo-antigen and increase the opportunity of cytotoxic T cells to recognize tumor cells, TMB (Tumor Mutational Burden, or Mutation load) and tumor neo-antigen load have previously been shown to profoundly influence the efficacy of anti-immune checkpoint therapy and could be independent predictors of ICI response in tumors, such as non-small cell lung cancer (Rizvi et al., 2015, 2018; Anagnostou et al., 2017). However, here we revealed that neither mutation load nor neo-antigen load, but NLRC5 expression could effectively predict the melanoma patients’ response to the immunotherapy. It may be partly explained by that only a small fraction of gene mutations occur on the cell surface protein and thus immunogenic (van Rooij et al., 2013; Yadav et al., 2014). In addition, mutation load and neo-antigen load appear independent of NLRC5 status in melanoma. The incorporation of NLRC5 expression and mutation/neo-antigen load into multivariable predictive models resulted in better predictive capability. Melanoma patients with both low NLRC5 expression and low mutation/neo-antigen load had the worst prognosis. This may be because such subgroup of melanoma has the weakest ability to induce/activate CD8^+^ T cells. The insignificant effect of immunotherapy in melanoma patients with low NLRC5 expression indicates that other treatments must be taken to achieve disease control. However, immunotherapy is effective in melanoma patients with high NLRC5 expression, which means that immunotherapy alone may achieve a good therapeutic effect. Our study is unable to distinguish NLRC5 downregulation of which cells in the tumor – infiltrating immune cells or melanoma cells themselves – has a greater contribution to the progression of melanoma and the response of immunotherapy, further work is needed to clarify this question. Nevertheless, our work suggests that the overall expression of NLRC5 in melanoma samples may be a potential new prognostic biomarker, especially for patients receiving immunotherapy. Second, the sample size of the current study to analyze the association between NLRC5 expression and immunotherapy response is not big, which may limit the effectiveness of the conclusion. In the future, more samples would need to be included to verify it. Third, our study showed that NLRC5 expression positively correlated with CD8^+^ T cell infiltration level in multiple melanoma datasets, but did not prove whether NLRC5 affects CD8^+^ T cell infiltration level in melanoma and its molecular mechanism.

In conclusion, our current work indicates that NLRC5 expression is regulated by SPI1 and methylation. Its low expression is an indicator for the malignant progression of melanoma and indicates poor immunotherapy treatment outcomes in melanoma patients. Our study is helpful to understand the intricate crosstalk between NLRC5 and immune regulation and may have implications for improving the immunotherapy of melanoma.

## Data Availability Statement

Publicly available datasets were analyzed in this study. This data can be found here: TCGA SKCM: https://xenabrowser.net/datapages/?cohort=TCGA%20Melanoma%20(SKCM)&removeHub=https%3A%2F%2Fxena.treehouse.gi.ucsc.edu%3A443; GSE54467: https://www.ncbi.nlm.nih.gov/geo/query/acc.cgi?acc=GSE54467; GSE59455: https://www.ncbi.nlm.nih.gov/geo/query/acc.cgi?acc=GSE59455; GSE65904: https://www.ncbi.nlm.nih.gov/geo/query/acc.cgi?acc=GSE65904; Van Allen et al. Science2015: https://www.cbioportal.org/study/summary?id=skcm_dfci_2015; Snyder et al. NEJM 2014: https://www.cbioportal.org/study/summary?id=skcm_mskcc_2014.

## Author Contributions

QY conceived and designed research. ZW, YZ, and NC collected the data. LL and QW analyzed the data. LL and QY wrote the manuscript. All authors read and approved the final manuscript.

## Conflict of Interest

The authors declare that the research was conducted in the absence of any commercial or financial relationships that could be construed as a potential conflict of interest.

## Footnotes

1 https://portals.broadinstitute.org/ccle

2 https://xenabrowser.net/datapages/?cohort=TCGA%20Melanoma%20(SKCM)

3 http://tide.dfci.harvard.edu

4 http://timer.comp-genomics.org

5 https://www.string-db.org/

6 http://humantfs.ccbr.utoronto.ca

## Supplementary Material

Supplementary Figure 1 SPI1 expression in melanoma. **(A,B)** SPI1 expression in single malignant melanoma cells, endothelial cells, CAFs (cancer-associated fibroblasts), T cells, B cells, macrophages, and NK Cells analyzed from melanoma datasets GEO72056 and GSE115978.

Supplementary Figure 2 NLRC5 expression correlates positively with SPI1 expression in melanoma receiving immunotherapy. **(A–F)** Spearman correlation analysis of the NLRC5 expression and SPI1 expression in the “Van Allen et al., Science 2015” dataset **(A)**, the “Snyder et al., NEJM 2014” dataset **(B)**, the “Riaz et al., Cell 2017” dataset **(C)**, the “Gide et al., Cancer cell 2019” dataset **(D,E)** and the “Lauss et al., Nat Commun 2017” datasets **(F)**, respectively. The type of immunotherapy received by melanoma patients in each dataset is shown. Spearman r and *p*-value for each correlation are shown.

Supplementary Figure 3 Prognostic value of SPI1 expression in melanoma. **(A–C)** Kaplan–Meier analysis of OS (overall survival), DSS (disease-specific survival), and PFI (progression-free interval) of patients with melanoma according to the SPI1 expression in TCGA SKCM dataset. **(D–F)** Kaplan–Meier analysis of OS (overall survival) of patients with melanoma according to the SPI1 expression in GSE54467 **(D)**, GSE53118 **(E)**, and GSE22153 **(F)** melanoma datasets, respectively. **(G–I)** Kaplan–Meier analysis of DSS (disease-specific survival) of patients with melanoma according to the SPI1 expression in GSE54467 **(G)**, GSE53118 **(H)**, and GSE65904 **(I)** melanoma datasets, respectively. The patients were stratified into high and low groups using the auto-select best cutoff determined by the R package “survminer.” Red: high SPI1 expression group, green: low SPI1 expression group.

Supplementary Figure 4 Mutation of NLRC5 in the “DFCI, Nature Medicine 2019” dataset. **(A)** Distribution of mutation sites in NLRC5 gene. **(B–D)** Effect of different NLRC5 mutations on HLA-A, HLA-B, and HLA-C expression.

Supplementary Figure 5 The correlation between NLRC5 expression and stromal/immune/estimate score in melanoma patients receiving immunotherapy. **(A–C)** Spearman correlation analysis of the NLRC5 expression and stromal score/immune score/estimate score in the “Van Allen et al. Science 2015” dataset, respectively. **(D–F)** Spearman correlation analysis of the NLRC5 expression and stromal score/immune score/estimate score in “Snyder et al. NEJM 2014” dataset, respectively.

Supplementary Figure 6 The correlation between NLRC5 expression and mutation/neoantigen load in melanoma patients receiving immunotherapy. **(A,B)** Spearman correlation analysis of the NLRC5 expression and mutation load/neoantigen load in the “Van Allen et al. Science 2015” dataset, respectively. **(C,D)** Spearman correlation analysis of the NLRC5 expression and mutation load/neoantigen load in the “Snyder et al. NEJM 2014” dataset, respectively.
