# Supplementary figures and images for: Clinical and Molecular Correlates of NLRC5 Expression in Patients With Melanoma
Source: Front Bioeng Biotechnol. 2021 Jul 9;9:690186. doi: 10.3389/fbioe.2021.690186 (PMC8299757; doi:10.3389/fbioe.2021.690186)

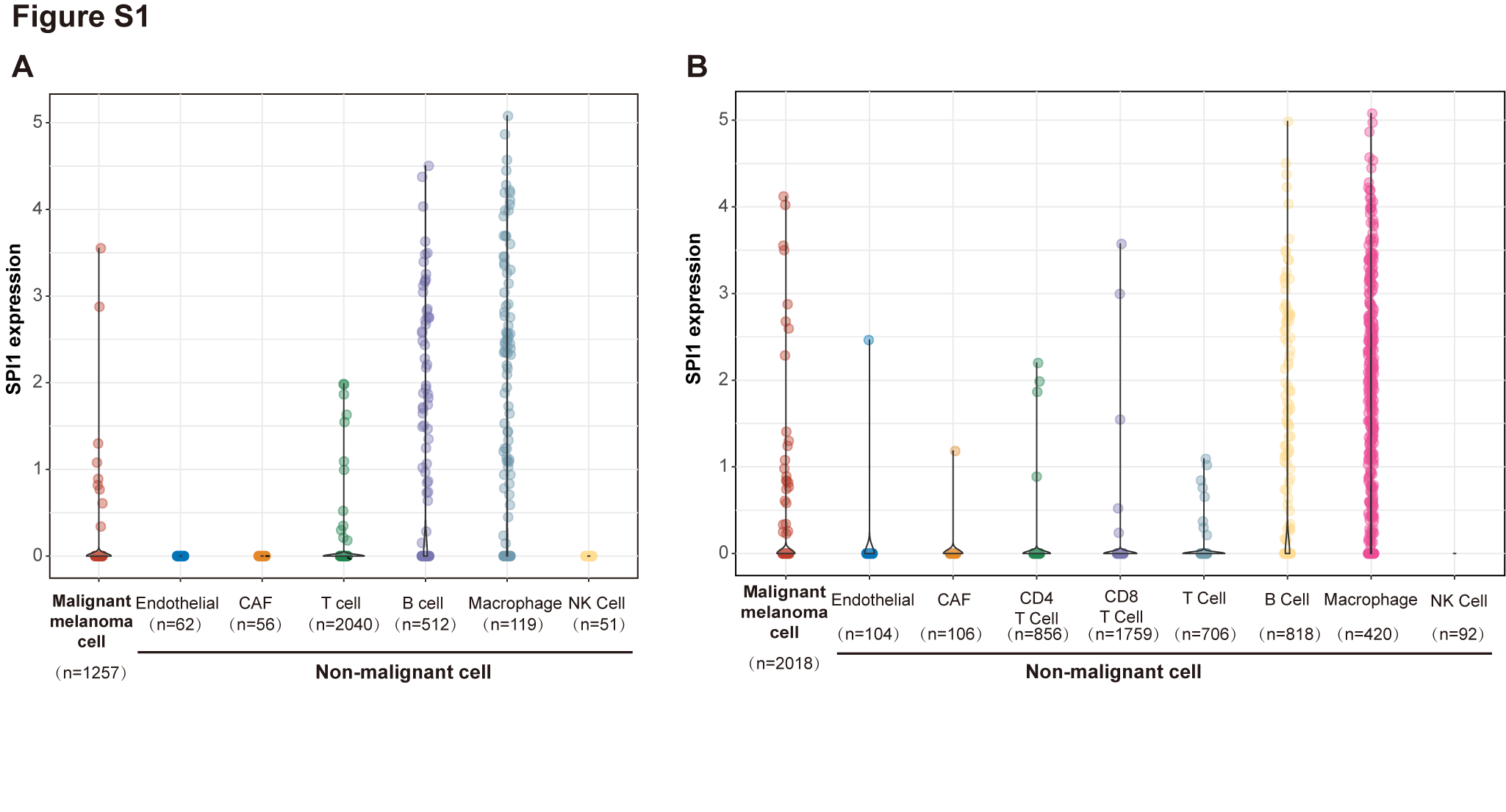

Supplement: Supplementary Figure 1 — SPI1 expression in melanoma. (A,B) SPI1 expression in single malignant melanoma cells, endothelial cells, CAFs (cancer-associated fibroblasts), T cells, B cells, macrophages, and NK Cells analyzed from melanoma datasets GEO72056 and GSE115978. [file Image_1.TIF]

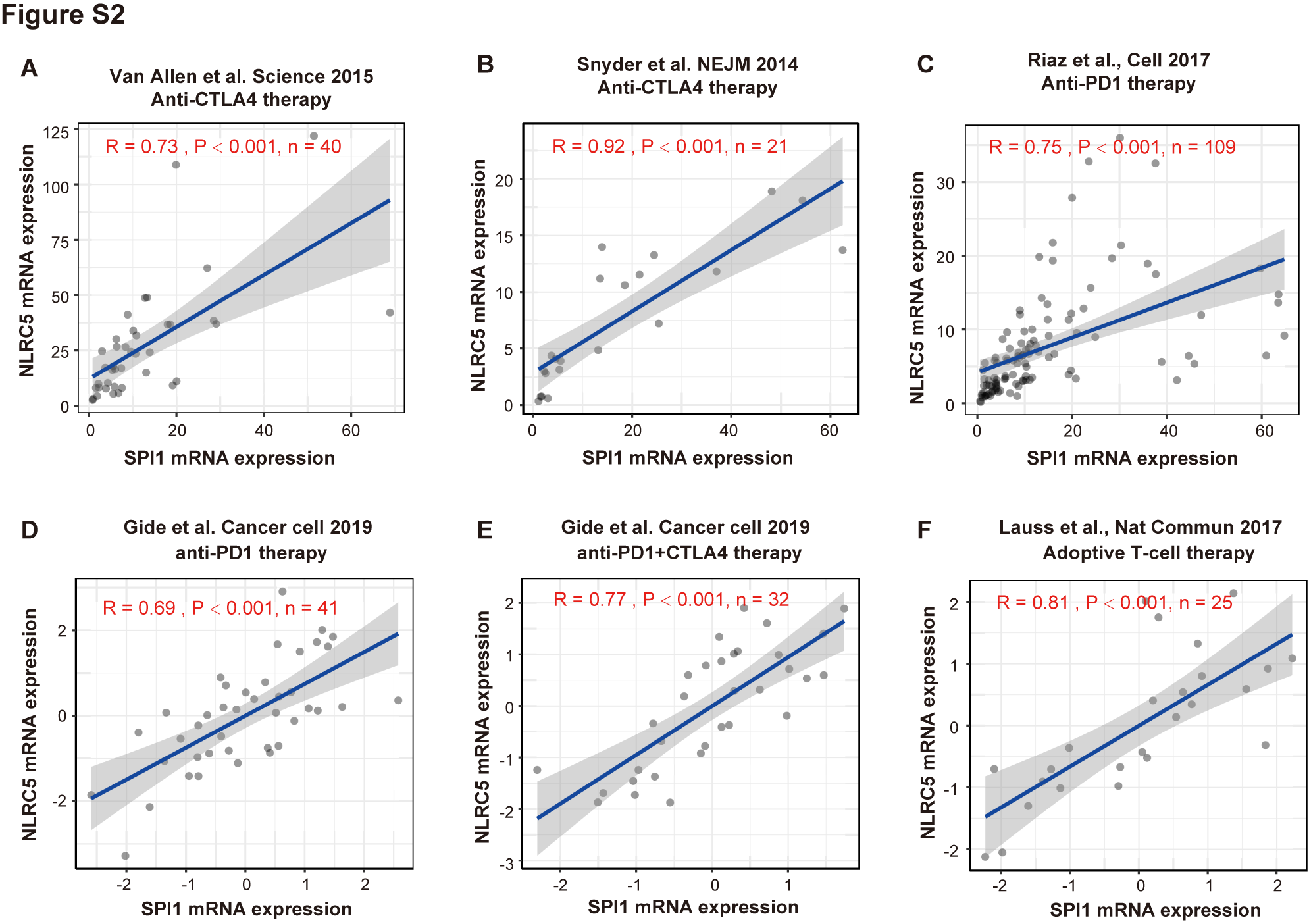

Supplement: Supplementary Figure 2 — NLRC5 expression correlates positively with SPI1 expression in melanoma receiving immunotherapy. (A–F) Spearman correlation analysis of the NLRC5 expression and SPI1 expression in the “Van Allen et al., Science 2015” dataset (A), the “Snyder et al., NEJM 2014” dataset (B), the “Riaz et al., Cell 2017” dataset (C), the “Gide et al., Cancer cell 2019” dataset (D,E) and the “Lauss et al., Nat Commun 2017” datasets (F), respectively. The type of immunotherapy received by melanoma patients in each dataset is shown. Spearman r and p-value for each correlation are shown. [file Image_2.TIF]

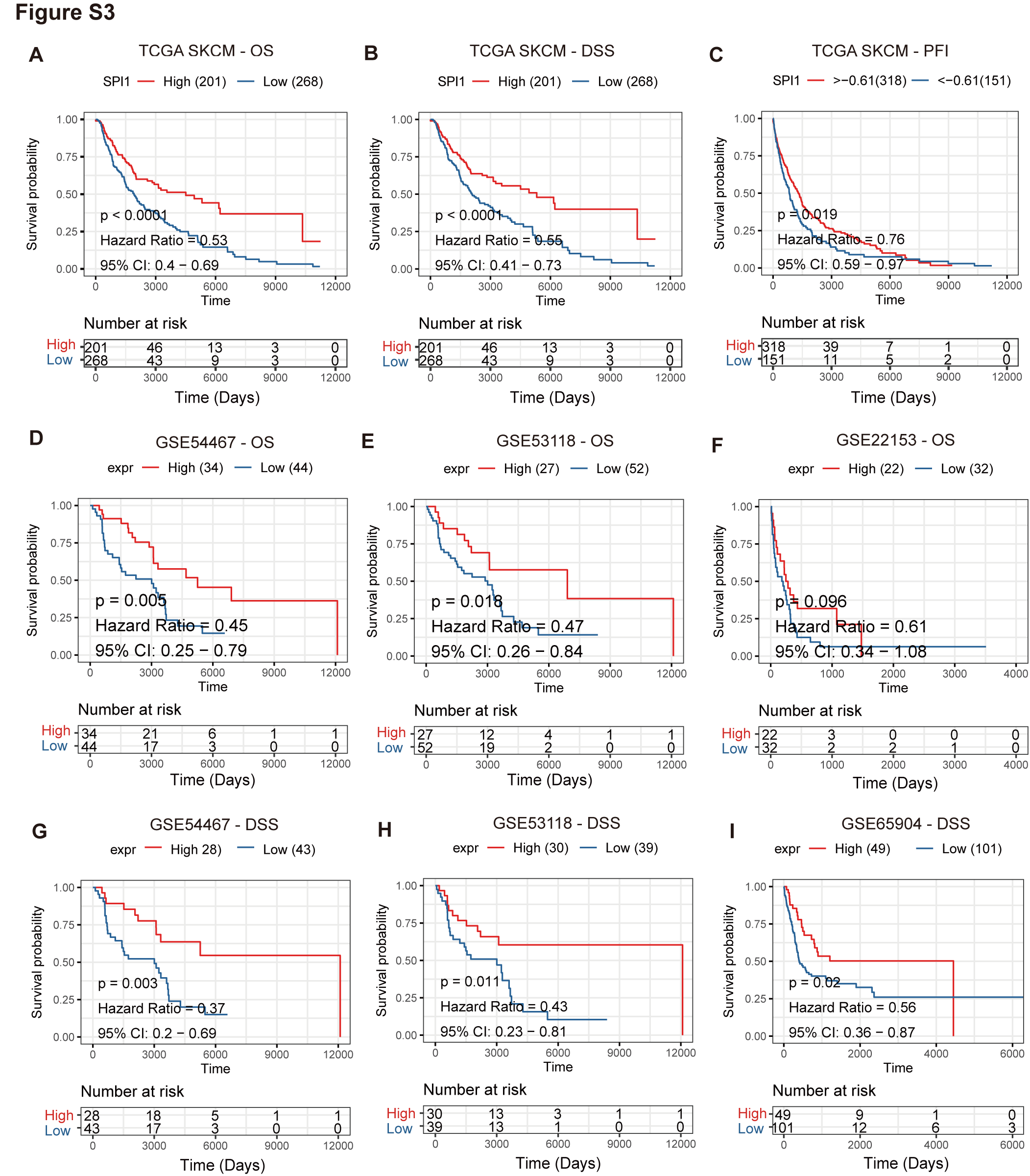

Supplement: Supplementary Figure 3 — Prognostic value of SPI1 expression in melanoma. (A–C) Kaplan–Meier analysis of OS (overall survival), DSS (disease-specific survival), and PFI (progression-free interval) of patients with melanoma according to the SPI1 expression in TCGA SKCM dataset. (D–F) Kaplan–Meier analysis of OS (overall survival) of patients with melanoma according to the SPI1 expression in GSE54467 (D), GSE53118 (E), and GSE22153 (F) melanoma datasets, respectively. (G–I) Kaplan–Meier analysis of DSS (disease-specific survival) of patients with melanoma according to the SPI1 expression in GSE54467 (G), GSE53118 (H), and GSE65904 (I) melanoma datasets, respectively. The patients were stratified into high and low groups using the auto-select best cutoff determined by the R package “survminer.” Red: high SPI1 expression group, green: low SPI1 expression group. [file Image_3.TIF]

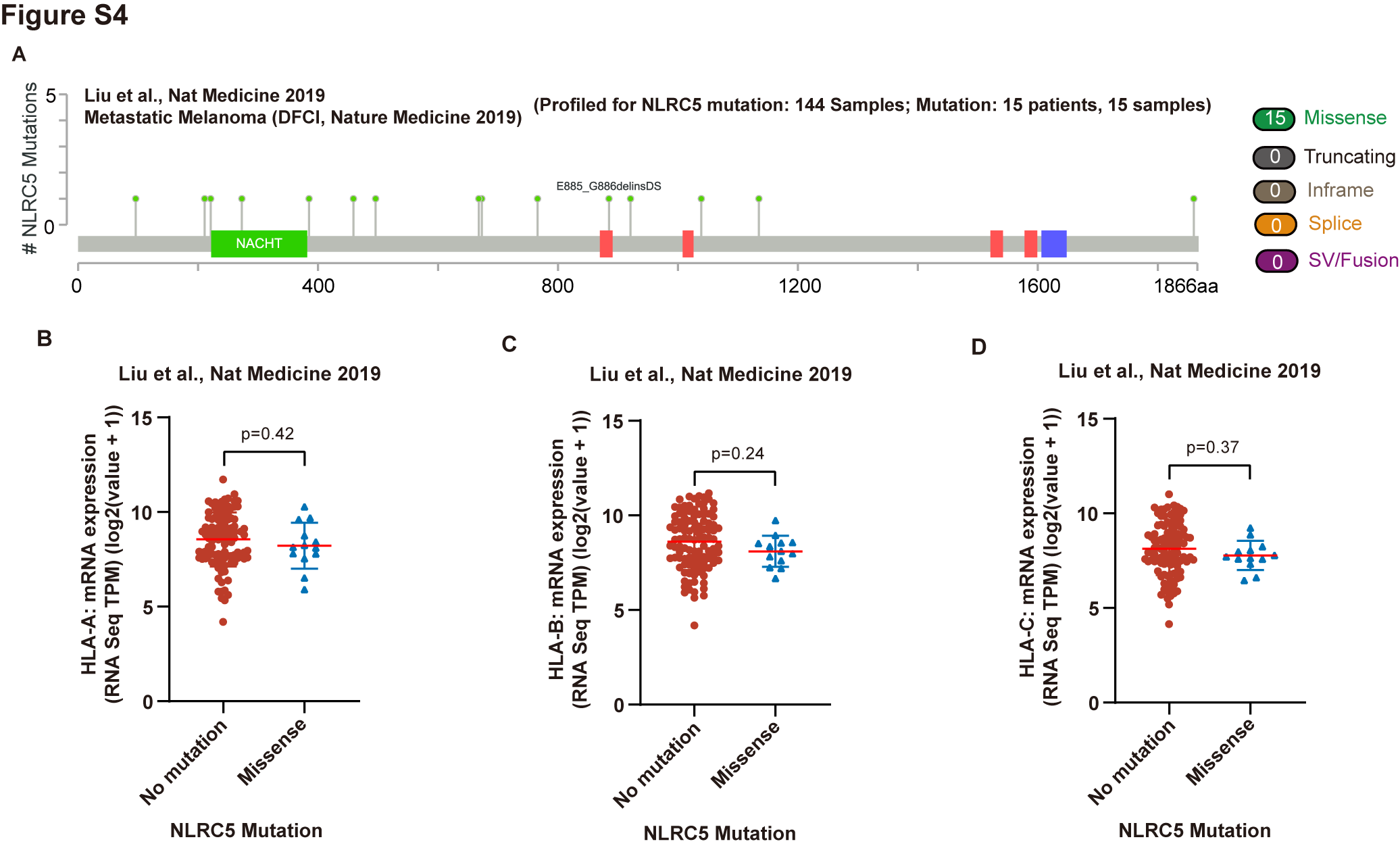

Supplement: Supplementary Figure 4 — Mutation of NLRC5 in the “DFCI, Nature Medicine 2019” dataset. (A) Distribution of mutation sites in NLRC5 gene. (B–D) Effect of different NLRC5 mutations on HLA-A, HLA-B, and HLA-C expression. [file Image_4.TIF]

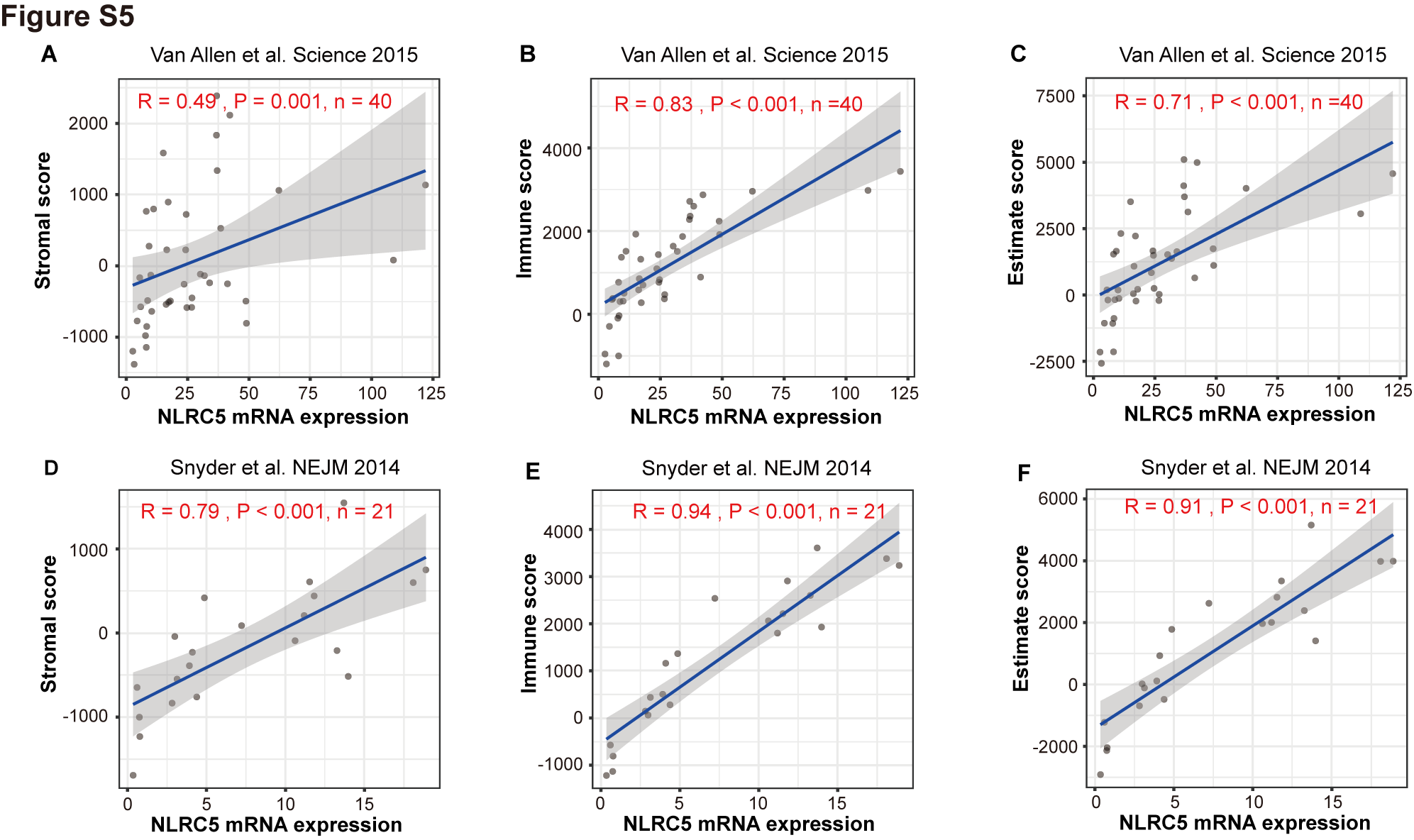

Supplement: Supplementary Figure 5 — The correlation between NLRC5 expression and stromal/immune/estimate score in melanoma patients receiving immunotherapy. (A–C) Spearman correlation analysis of the NLRC5 expression and stromal score/immune score/estimate score in the “Van Allen et al. Science 2015” dataset, respectively. (D–F) Spearman correlation analysis of the NLRC5 expression and stromal score/immune score/estimate score in “Snyder et al. NEJM 2014” dataset, respectively. [file Image_5.TIF]

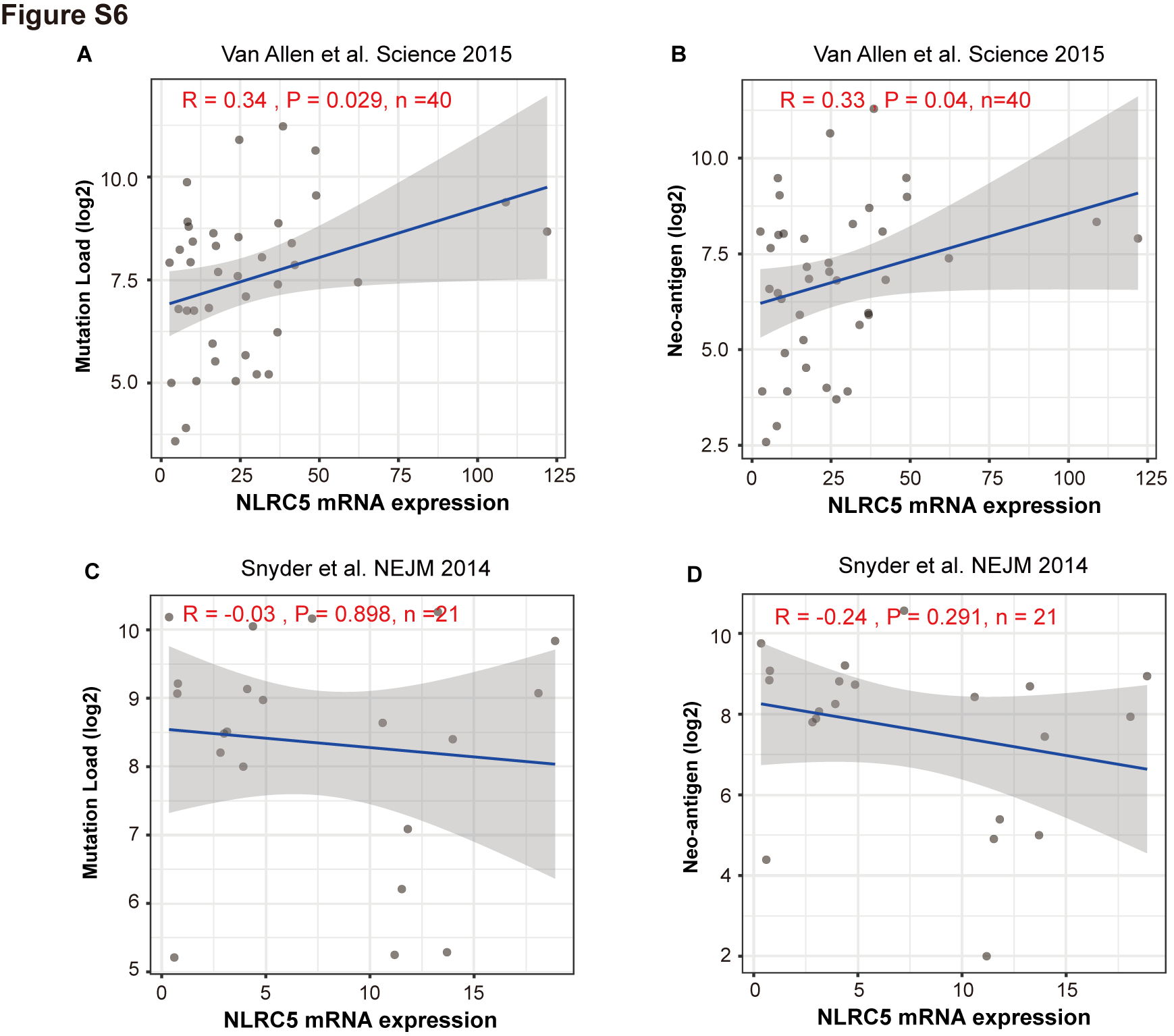

Supplement: Supplementary Figure 6 — The correlation between NLRC5 expression and mutation/neoantigen load in melanoma patients receiving immunotherapy. (A,B) Spearman correlation analysis of the NLRC5 expression and mutation load/neoantigen load in the “Van Allen et al. Science 2015” dataset, respectively. (C,D) Spearman correlation analysis of the NLRC5 expression and mutation load/neoantigen load in the “Snyder et al. NEJM 2014” dataset, respectively. [file Image_6.TIF]
