# Supplementary material for: Clinical and Molecular Correlates of NLRC5 Expression in Patients With Melanoma
Source: Front Bioeng Biotechnol. 2021 Jul 9;9:690186. doi: 10.3389/fbioe.2021.690186 (PMC8299757; doi:10.3389/fbioe.2021.690186)
Supplement: Supplementary file 7 [file Table_1.DOCX]

**Table S1. The top 15 transcription factors positively correlated with NLRC5 expression in TCGA SKCM.**

| Cancer | Coefficient-R | p-value |
| --- | --- | --- |
| IRF1 | 0.875426467 | <0.0001 |
| TBX21 | 0.834385914 | <0.0001 |
| ETV7 | 0.789293362 | <0.0001 |
| ZBED2 | 0.782409521 | <0.0001 |
| SP140 | 0.774336667 | <0.0001 |
| ASCL2 | 0.767744754 | <0.0001 |
| STAT1 | 0.767099715 | <0.0001 |
| BCL11B | 0.760287061 | <0.0001 |
| IRF8 | 0.744422569 | <0.0001 |
| ZBTB32 | 0.742401872 | <0.0001 |
| ZNF831 | 0.721063986 | <0.0001 |
| ZNF683 | 0.718867896 | <0.0001 |
| SPI1 | 0.717244914 | <0.0001 |
| IRF9 | 0.707340467 | <0.0001 |
| STAT4 | 0.695960573 | <0.0001 |
