# Supplementary material for: Clinical and Molecular Correlates of NLRC5 Expression in Patients With Melanoma
Source: Front Bioeng Biotechnol. 2021 Jul 9;9:690186. doi: 10.3389/fbioe.2021.690186 (PMC8299757; doi:10.3389/fbioe.2021.690186)
Supplement: Supplementary file 8 [file Table_2.DOCX]

**Table S2. Correlation analysis for SPI1 and NLRC5 mRNA expression in TCGA.**

| Cancer | Cancer Full Name | Sample Number | Coefficient-R | p-value |
| --- | --- | --- | --- | --- |
| TGCT | Testicular Germ Cell Tumors | 156 | 0.782 | 1.91E-33 |
| THCA | Thyroid Carcinoma | 510 | 0.724 | 5.82E-84 |
| SKCM | Skin Cutaneous Melanoma | 471 | 0.704 | 1.32E-71 |
| UVM | Uveal Melanoma | 80 | 0.694 | 9.13E-13 |
| LGG | Brain Lower Grade Glioma | 529 | 0.681 | 1.82E-73 |
| BLCA | Bladder Urothelial Carcinoma | 411 | 0.669 | 1.30E-54 |
| PRAD | Prostate Adenocarcinoma | 499 | 0.611 | 2.71E-52 |
| BRCA | Breast Invasive Carcinoma | 1104 | 0.591 | 4.04E-105 |
| LIHC | Liver Hepatocellular Carcinoma | 374 | 0.571 | 1.10E-33 |
| THYM | Thymoma | 119 | 0.57 | 1.27E-11 |
| LUSC | Lung Squamous Cell Carcinoma | 501 | 0.543 | 8.94E-40 |
| CESC | Cervical Squamous Cell Carcinoma and Endocervical Adenocarcinoma | 306 | 0.535 | 4.61E-24 |
| PAAD | Pancreatic Adenocarcinoma | 178 | 0.532 | 2.14E-14 |
| OV | Ovarian Serous Cystadenocarcinoma | 379 | 0.514 | 5.70E-27 |
| UCS | Uterine Carcinosarcoma | 56 | 0.514 | 5.04E-05 |
| PCPG | Pheochromocytoma and Paraganglioma | 183 | 0.475 | 1.09E-11 |
| LUAD | Lung Adenocarcinoma | 526 | 0.449 | 1.95E-27 |
| ESCA | Esophageal Carcinoma | 162 | 0.446 | 2.71E-09 |
| KIRP | Kidney Renal Papillary Cell Carcinoma | 289 | 0.419 | 1.06E-13 |
| UCEC | Uterine Corpus Endometrial Carcinoma | 548 | 0.412 | 7.97E-24 |
| MESO | Mesothelioma | 86 | 0.411 | 8.57E-05 |
| HNSC | Head and Neck Squamous Cell Carcinoma | 502 | 0.399 | 1.19E-20 |
| CHOL | Cholangiocarcinoma | 36 | 0.386 | 2.01E-02 |
| KIRC | Kidney Renal Clear Cell Carcinoma | 535 | 0.373 | 4.41E-19 |
| SARC | Sarcoma | 263 | 0.362 | 1.43E-09 |
| COAD | Colon Adenocarcinoma | 471 | 0.353 | 2.85E-15 |
| STAD | Stomach Adenocarcinoma | 375 | 0.326 | 9.65E-11 |
| READ | Rectum Adenocarcinoma | 167 | 0.325 | 1.83E-05 |
| ACC | Adrenocortical Carcinoma | 79 | 0.188 | 9.77E-02 |
| KICH | Kidney Chromophobe | 65 | 0.079 | 5.29E-01 |
