# Supplementary material for: Clinical and Molecular Correlates of NLRC5 Expression in Patients With Melanoma
Source: Front Bioeng Biotechnol. 2021 Jul 9;9:690186. doi: 10.3389/fbioe.2021.690186 (PMC8299757; doi:10.3389/fbioe.2021.690186)
Supplement: Supplementary file 11 [file Table_5.DOCX]

**Table S5. Correlation between NLRC5 expression and immune cell infiltration estimated by “TIMER” in melanoma datasets**.

|  | TCGA SKCM | |  | GSE54467 | |  | GSE59455 | |  | GSE65904 | |
| --- | --- | --- | --- | --- | --- | --- | --- | --- | --- | --- | --- |
|  | Spearman r | P value |  | Spearman r | P value |  | Spearman r | P value |  | Spearman r | P value |
| B cell | 0.334 | <0.0001 |  | 0.627 | <0.0001 |  | 0.4085 | <0.0001 |  | 0.413 | <0.0001 |
| CD4+ T cell | 0.6296 | <0.0001 |  | 0.524 | <0.0001 |  | 0.1254 | 0.1383 |  | 0.1825 | 0.0074 |
| CD8+ T cell | 0.5088 | <0.0001 |  | 0.5074 | <0.0001 |  | 0.4856 | <0.0001 |  | 0.5119 | <0.0001 |
| Neutrophil | 0.6727 | <0.0001 |  | 0.2821 | 0.0118 |  | 0.338 | <0.0001 |  | 0.2629 | <0.0001 |
| Macrophage | 0.2485 | <0.0001 |  | 0.2719 | 0.0154 |  | 0.04035 | 0.6348 |  | 0.06665 | 0.3319 |
| Dendritic cell | 0.7163 | <0.0001 |  | 0.65 | <0.0001 |  | 0.5156 | <0.0001 |  | 0.5587 | <0.0001 |
